# Group 13 Element Trihalide Complexes of Anionic N‐Heterocyclic Carbenes

**DOI:** 10.1002/asia.201901774

**Published:** 2020-02-20

**Authors:** Luong Phong Ho, Lisa Anders, Matthias Tamm

**Affiliations:** ^1^ Institut für Anorganische und Analytische Chemie Technische Universität Braunschweig Hagenring 30 38106 Braunschweig Germany

**Keywords:** N-heterocyclic carbenes, boron, aluminium, gallium, indium

## Abstract

A series of group 13 complexes of the general type [{(WCA‐IDipp)EX_3_}Li(solv)] (E=B, Al, Ga, In; X=Cl, Br) that bear an anionic N‐heterocyclic carbene ligand with a weakly coordinating borate moiety (WCA‐IDipp, WCA=B(C_6_F_5_)_3_ and IDipp=1,3‐bis(2,6‐diisopropylphenyl)imidazolin‐2‐ylidene) were prepared by the reaction of the respective group 13 trihalides (EX_3_) with the lithium salt [(WCA‐IDipp)Li ⋅ toluene]. The molecular structures of the BBr_3_, AlCl_3_, AlBr_3_, GaCl_3_ and InCl_3_ adducts were established by X‐ray diffraction analyses, revealing the formation of coordination polymers linked by halide‐lithium interactions, except for the indium derivative, which consists of isolated [Li(THF)_4_]^+^ and [(WCA‐IDipp)InCl_3_]^−^ ions in the solid state.

## Graphical abstract

**Expanding the library**: Several group 13 complexes of the type [((WCA‐IDipp)EX_3_)Li] (E=B, Al, Ga and In; X=Cl, Br) that bear anionic *N*‐heterocyclic carbene ligands with a weakly coordinating borate moiety were prepared.

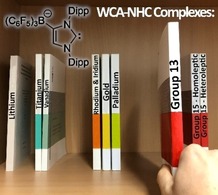

## Introduction

**Figure 1:**
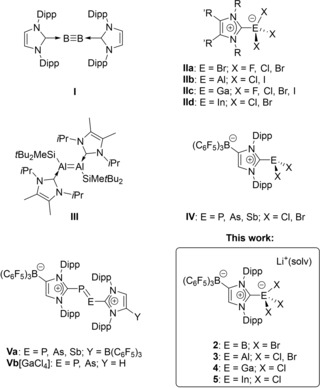
Selected group 13 and 15 compounds bearing N‐heterocyclic carbenes (Dipp=2,6‐diisopropylphenyl; R=alkyl, aryl; R’=H, alkyl).

The first synthesis and isolation of a stable crystalline N‐heterocyclic carbene (NHC) by Arduengo and coworkers in 1991^1^, ^2^, ^3^ initiated the extensive development of new main group element‐carbene adducts.^4^, ^5^ Especially the strong σ donating, and partially also the π accepting properties of NHCs proved to be very useful for the stabilization of highly reactive species, and consequently, this class of ligands has become one of the most important in modern organometallic chemistry.^6^, ^7^, ^8^, ^9^ For instance, their ability to stabilize unusual low valent main group element compounds could be demonstrated by the isolation of [(IDipp)_2_B_2_] (**I**, IDipp=1,3‐bis(2,6‐diisopropylphenyl)imidazolin‐2‐ylidene, Figure 1),^10^ which contains a boron‐boron triple bond and represents one of the many “carbene‐stabilized main group diatomic allotropes”.^11^ Remarkably, direct access to **I** by reductive coupling of [(IDipp)BBr_3_] with KC_8_ was not possible, and a stable neutral diborane containing a B=B double bond was isolated instead.^12^ Nevertheless, a large variety of [(NHC)BX_3_] complexes (**IIa**, X=F, Cl, Br) have been prepared during the last 20 years and have served as starting materials for numerous transformations.^7^, ^13^, ^14^, ^15^, ^16^ Furthermore, the heavier congeners [(NHC)AlX_3_] (**IIb**, X=Cl, I),^17^, ^18^, ^19^, ^20^, ^21^ [(NHC)GaX_3_] (**IIc**, X=F, Cl, Br, I)^22^, ^23^, ^24^, ^25^, ^26^ and [(NHC)InX_3_] (**IId**, X=Cl, Br)^27^, ^28^, ^29^ were also synthesized, and for aluminum, the Al=Al double bonded species [(^Me^I*i*Pr)_2_Al_2_(SiMe*t*Bu_2_)_2_] (**III**, ^Me^I*i*Pr=1,3‐bis(isopropyl)‐4,5‐dimethylimidazolin‐2‐ylidene) bearing two NHCs was also reported.^30^ Very recent examples also include the stabilization of group 13–15 complexes such as [(NHC)B(H) =N(H)⋅BAr^F^_3_],^31^ [(NHC)BH_2_PH_2_]^32^ and [(NHC)EH_2_PH_2_] (E=Al, Ga).^33^

**Scheme 1:**
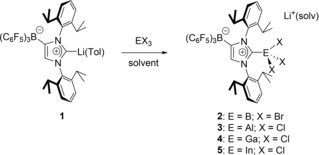
Preparation of group 13 adducts with the weakly coordinating anionic N‐heterocyclic carbene WCA‐IDipp.

Anionic N‐heterocyclic carbenes represent another emerging class of ancillary ligands,^34^ and our group has developed WCA‐NHC ligands that contain a weakly coordinating anionic (WCA) borate moiety in the 4‐position of the N‐heterocycle. For instance, the lithium salt [(WCA‐IDipp)Li ⋅ toluene] (**1**, Scheme 1) was used as a carbene transfer reagent for the synthesis of transition metal complexes and homogenous (pre‐)catalysts.^35^, ^36^, ^37^, ^38^, ^39^ More recently, this type of anionic carbenes made their first appearance in main group element chemistry, and the group 15 complexes [(WCA‐IDipp)E'X_2_] (**IV**, E’=P, As, Sb; X=Cl, Br) were used as starting materials for the synthesis of the neutral dipnictenes **Va**.^40^ Furthermore, complexes **IV** were also used to establish hetero‐dielement bonds by employing a modular approach,^41^, ^42^ which involved the reaction of **IV** with the carbene‐phosphinidene adduct (IDipp)PSiMe_3_ 43 under Me_3_SiCl elimination. This route provided heteroleptic diphosphorus and arsenic‐phosphorus systems bearing both an anionic and a neutral NHC such as the cationic diphosphene and diarsene species **Vb** together with the corresponding neutral radicals.^44^ In order to expand the library of potential substrates for the preparation of mixed dielement species, we aimed at the synthesis of group 13 element WCA‐NHC complexes and have isolated compounds **2–5**, which are described in this contribution.

## Results and Discussion

The reactions of [(WCA‐IDipp)Li ⋅ toluene] (**1**) with the group 13 trihalides EX_3_ were performed by stirring in toluene (for E=B, Al, Ga) or THF (for E=In) solution for 1–12 h under ambient conditions, affording the ate‐complexes [{(WCA‐IDipp)EX_3_}Li(solv)] (**2**: E=B, X=Br; **3**: E=Al, X=Cl; **4**: E=Ga, X=Cl; **5**: E=In, X=Cl) as highly air and moisture sensitive colorless solids in good yields (48–86 %, Scheme 1). It should be noted that the solubility of these complexes in toluene decreases in the order B>Al>Ga>In. Surprisingly, elimination of lithium chloride or bromide from compounds **2**–**5** to form complexes of the type [(WCA‐IDipp)EX_2_] was not observed, despite the potential of the WCA‐NHC ligand to stabilize a vacant coordination site by intramolecular arene coordination as previously found for transition metal complexes.^36^, ^38^

All new complexes were characterized by ^1^H, ^11^B, ^13^C and ^19^F NMR spectroscopy in THF‐*d*_8_ solution (see the Supporting Information for the presentation of all spectra). The structural resemblance of **2**–**5** results in similar splitting patterns observed for the ^1^H NMR signals. In general, the signals corresponding to the two Dipp substituents are split into two sets because of the loss of symmetry introduced by the borate moiety in the backbone. The characteristic singlet for the backbone CH hydrogen atoms is found at 7.05 ppm in case of the BBr_3_ derivative **2**. For the chloride derivatives **3**–**5**, this signal shifts from 6.65 ppm (**3**, E=Al) to 6.73 ppm (**4**, E=Ga) and 6.86 ppm (**5**, E=In). In the ^13^C NMR spectra of **2** and **3**, the signal corresponding to the C_Carbene_ atom could not be resolved, whereas this signal is found at 158.1 and 174.2 ppm for **4** and **5**, respectively. For all compounds, the ^11^B resonance for the boron atom in the backbone is found almost invariably as a sharp singlet at ca. −15.7 ppm and the boron atom of the BBr_3_ moiety in compound **2** can be found at 21.6 ppm as a broad singlet. The ^19^F NMR spectra show three signals for the *ortho*‐, *meta*‐ and *para*‐fluorine atoms.

**Figure 2:**
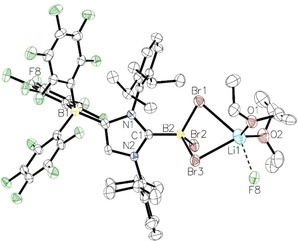
Molecular structure of **2** ⋅ 2 Et_2_O with thermal displacement parameters drawn at 50 % probability; hydrogen atoms are omitted for clarity; pertinent structural data of compounds **2–5** are assembled in Table 1.

**Figure 3:**
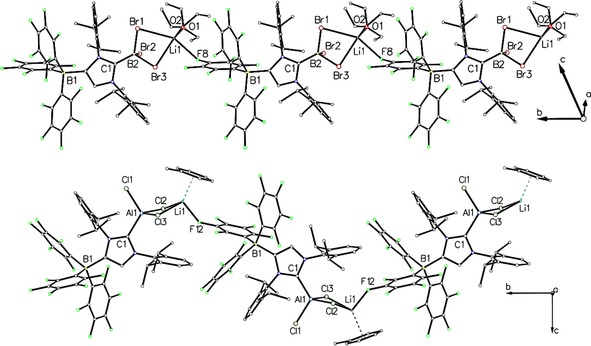
Top: Chain polymer of **2** ⋅ 2 Et_2_O. Hydrogen atoms are omitted for clarity. The overall chain direction is parallel to the b axis. Bottom: Chain polymer of **3** ⋅ 2 toluene. Hydrogen atoms and the second toluene molecule are omitted for clarity. The overall chain direction is parallel to the b axis and the view direction is along the a axis.

The molecular structure of **2** was determined by X‐Ray diffraction analysis of a single crystal (refined as a two‐component twin) obtained from a saturated Et_2_O solution cooled to −40 °C (Figure 2). Compound **2** ⋅ 2 Et_2_O crystallizes in the space group *P* $\bar{1}$ , and the C_Carbene_−B bond length is 1.624(7) Å, which falls in the range found for C−B single bonds at a four‐coordinate boron site (Table 1).^45^ This distance is also identical within experimental error to that found in the neutral NHC adduct [(IDipp)BBr_3_] (1.623(7) Å).^12^ Two of the bromine atoms bound to the boron atom display similar B–Br bond lengths (B2−Br1=2.023(5) Å, B2−Br2=2.013(6) Å), whereas the B2−Br3 bond is slightly longer (2.065(7) Å). Compound **2** ⋅ 2 Et_2_O crystallizes as a coordination polymer along the b axis, in which the lithium ion is coordinated by two of the bromine atoms from the same boron atom, two diethyl ether molecules and one of the *para*‐fluorine atoms of the borate moiety in the next asymmetric unit (Figure 3). The Br−Li distances are 2.803(13) (Li1−Br1) and 2.592(11) Å (Li1−Br3); the shorter Li−Br3 distance is consistent with the longer B2−Br3 bond lengths of 2.065(7) Å (*vide supra*). The Li1−F8 distance is 2.425(14) Å, and the Li−O distances are 1.894(13) and 1.904(11) Å, respectively.

**Table 1:** Selected bond lengths [Å] and angles [°] in compounds **2–5**.

**E**	**X**	**C–E**	**E–X**	**N–C–N**
B	Br	1.624(7)	2.013(6) 2.023(5) 2.065(7)	105.6(4)
Al	Cl	2.019(2)	2.1152(7) 2.1626(8) 2.1723(8)	104.73(15)
Al^[a]^	Br	2.019(2)	2.2737(8) 2.3034(9) 2.3265(8)	104.68(18)
Ga	Cl	1.983(2)	2.1851(8) 2.2003(7) 2.2316(8)	105.64(18)
In	Cl	2.210(3)	2.3541(18) 2.3544(11) 2.3683(16)	106.2(3)

[a] Molecular structure can be found in the Supporting Information.

**Figure 4:**
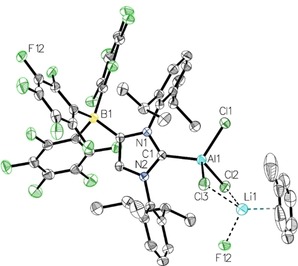
Molecular structure of **3** ⋅ 2 toluene with thermal displacement parameters drawn at 50 % probability; hydrogen atoms and the second toluene molecule are omitted for clarity; pertinent structural data of compounds **2**–**5** are assembled in Table 1.

The aluminium derivative crystallized from a saturated solution of [{(WCA‐IDipp)AlCl_3_}Li] (**3**) in toluene layered with *n*‐hexane at room temperature. A single crystal of **3** ⋅ 2 toluene obtained by this method was subjected to X‐ray diffraction analysis, and the resulting molecular structure is presented in Figure 4. **3** ⋅ 2 toluene crystallizes in the space group *P*2_1_/*c*, and the C1−Al1 distance is 2.019(2) Å, which is in excellent agreement with the C−Al bond length of 2.017(2) Å in [(IMes)AlCl_3_] (Table 1).^17^ **3** ⋅ 2 toluene forms a coordination polymer along the b axis; the lithium counterion is bound to two chlorine atoms, one *para*‐fluorine atom in the borate moiety of the next asymmetric unit and one toluene molecule, which affords a three‐legged piano‐stool geometry (Figure 3). The Al−Cl bond lengths are 2.1152(7) Å (Al1−Cl1), 2.1626(8) Å (Al1−Cl2) and 2.1723(8) Å (Al1−Cl3), with the latter two showing the expected elongation because of lithium coordination (Li1−Cl2=2.467(5) Å, Li1−Cl3=2.451(4) Å). The toluene ligand binds to Li1 in a symmetric η^6^‐fashion with lithium‐carbon distances in the range of 2.460–2.552 Å, and the Li1−F12 distance is 1.935(4) Å. It should be noted that the molecular structure of the aluminium bromide derivative [{(WCA‐IDipp)AlBr_3_}Li] ⋅ 2 toluene was also determined by X‐ray diffraction analysis. The molecular structure together with relevant data can be found in the Supporting Information.

**Figure 5:**
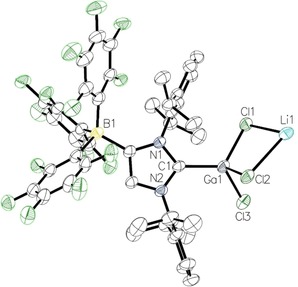
Molecular structure of **4** ⋅ C_6_D_6_ with thermal displacement parameters drawn at 50 % probability; hydrogen atoms and one benzene molecule are omitted for clarity; pertinent structural data of compounds **2**–**5** are assembled in Table 1.

Single crystals of gallium complex **4** ⋅ C_6_D_6_ were obtained by slow evaporation of a C_6_D_6_ solution at ambient temperature under inert conditions and subjected to X‐ray diffraction analysis. **4** ⋅ C_6_D_6_ crystallizes in the space group *P*2_1_/*c*, and the resulting molecular structure is shown in Figure 5. The C1−Ga1 bond length of 1.983(2) Å (Table 1) is slightly shorter compared to [(IDipp)GaCl_3_] (2.015(2) Å),^23^ and it is also shorter than the bond length found for C1−Al1 in complex **3** (2.019(2) Å). This can be attributed to the similar covalent and van der Waals radii of the aluminium and gallium atoms.^46^, ^47^ Similar observations were made for hydride complexes [(IDipp)AlH_3_] (2.0556(13) Å)^29^ and [(IDipp)GaH_3_] (2.0545(14) Å).^48^

**Figure 6:**
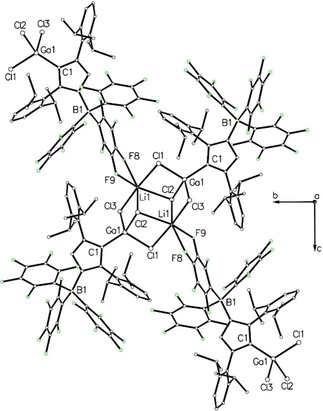
Coordination‐polymer of **4** ⋅ C_6_D_6_. Hydrogen atoms and the C_6_D_6_ molecule are omitted for clarity. The overall polymer expansion is within the bc plane and the view direction is along the a axis.

In contrast to the 1D coordination polymers observed in the packing of **2** ⋅ 2 Et_2_O and **3** ⋅ 2 toluene, **4** ⋅ C_6_D_6_ forms a 2D coordination polymer in the solid state, stretched out in the bc plane (Figure 6). The gallium atom displays distorted tetrahedral geometry with markedly different Ga−Cl bond lengths (Table 1) of 2.1851(8) Å (Ga1−Cl1), 2.2003(7) Å (Ga1−Cl3) and 2.2316(8) Å (Ga1−Cl2), since the chlorine atoms are bridging the gallium and lithium atoms in μ_2_‐ (Cl1 and Cl3) and μ_3_‐fashion (Cl2). This affords centrosymmetric Ga_2_Li_2_Cl_6_ clusters, in which the lithium atoms show distorted octahedral geometries by interaction with four chlorine atoms and two fluorine atoms of the borate moiety. The two disparate Li−F distances of 2.077(5) Å (Li1−F9) and 2.500(5) Å (Li1−F8) indicate that the Li coordination sphere can also be described as square‐pyramidal with the weakly bound F8 atom filling the vacant equatorial position.

**Figure 7:**
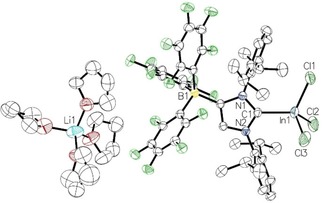
Molecular structure of **5** ⋅ 4 THF with thermal displacement parameters drawn at 50 % probability; hydrogen atoms are omitted for clarity; pertinent structural data of compounds **2**–**5** are assembled in Table 1.

Single crystals of the indium complex **5** ⋅ 4 THF were obtained from THF solution layered with *n*‐hexane, and X‐ray diffraction analysis revealed the separation of the fully solvated [Li(THF)_4_]^+^ cation from the [(WCA‐IDipp)InCl_3_]^−^ anion (Figure 7). The lithium ion is coordinated by the THF molecules in a tetrahedral fashion. The C1−In1 bond length is 2.210(3) Å (Table 1), which is in good agreement with the values established for [(IMes)InCl_3_] (2.200(7) Å) and [(IDipp)InBr_3_] (2.212(8) Å).^28^, ^29^ The indium atom displays a fairly distorted tetrahedral geometry; since the chlorine atoms are devoid of any relevant secondary interaction, similar indium‐chlorine bonds of 2.3541(18) Å (In1−Cl1), 2.3544(11) Å (In1−Cl2) and 2.3683(16) Å (In1−Cl3) are observed.

## Conclusion

The reaction of the lithium carbene complex [(WCA‐IDipp)Li ⋅ toluene] (**1**) with group 13 element trihalides afforded in all cases ate‐complexes of the type [(WCA‐IDipp)EX_3_]^−^ (E=B, Al, Ga, In; X=Cl, Br), and elimination of lithium chloride or bromide was not observed. Accordingly, these complexes are anionic analogues of related adducts with neutral N‐heterocyclic carbenes (NHC). However, the lithium counterion in the complexes **2**–**5** determines the solid‐state structure by linking the anionic carbene complexes or by formation of solvent‐separated ion pairs. With the compounds **2**–**5** in hand, we intend to further study their use as starting materials for the formation of group 13 compounds with element‐element (multiple) bonds under reducing conditions. Alternatively, their combination with NHC‐phosphinidene and arsinidene adducts of the type (NHC)ESiMe_3_ (E=P, As)^41^, ^42^, ^44^, ^49^ might afford group 13/15 hetero‐dielement compounds as new additions to the growing family of carbene‐stabilized diatomic species.

## Experimental Section

### Materials and Methods

Unless otherwise indicated, all starting materials were obtained from commercial sources (Sigma‐Aldrich, Alfa‐Aesar, Roth, TCI, VWR or Fisher Chemical) and were used without further purification. Elemental analyses were carried out on a Vario Micro Cube System. All operations with air‐ and moisture‐sensitive compounds were performed in a glove box under a dry argon atmosphere (MBraun 200B) or on a high vacuum line using Schlenk techniques. The ^1^H, ^11^B, ^13^C and ^19^F NMR spectra were recorded on Bruker AVII300 (300 MHz), Bruker AVIIHD500 (500 MHz) and Bruker AVII600 (600 MHz). The chemical shifts are expressed in parts per million (ppm) with the residual solvent signal as internal standard for ^1^H and ^13^C NMR spectra. All other spectra were calibrated using external references. Coupling constants (*J*) are reported in Hertz (Hz) and splitting patterns are indicated as s (singlet), d (doublet), t (triplet), sept (septet), m (multiplet) and br (broad). ^11^B, ^13^C and ^19^F NMR spectra were measured broadband proton decoupled. Presentations of all NMR spectra can be found in the Supporting Information. *n*‐Hexane, tetrahydrofuran (THF), diethyl ether (Et_2_O), and toluene were purified by distillation over sodium/benzophenone. Deuterated solvents were purified by stirring the degassed solvents with Na/K alloy overnight. Subsequently, the solvents were filtered and then distilled under reduced pressure. All solvents were stored over molecular sieves (4 Å) in argon atmosphere prior to use. [(WCA‐IDipp)Li ⋅ toluene] was prepared according to a literature procedure.^36^ For crystallographic details, see the Supporting Information.

### Synthetic Procedures

[{(WCA‐IDipp)BBr_3_}Li] (**2**). BBr_3_ (250 mg, 1 mmol, 1 eq.) was dissolved in toluene (40 mL) and [(WCA‐IDipp)Li ⋅ toluene] (**1**, 1 g, 1 mmol, 1 eq.) was added slowly as a solid in small portions. The resulting cloudy solution was stirred for 5 h at room temperature, affording a pale‐yellow suspension, which was filtered over Celite. The resulting yellow solution was evaporated and the resulting residue was washed with *n*‐hexane (3×5 mL). Residual solvent was removed in vacuo, yielding the product as a colorless solid (841 mg, 0.787 mmol, 79 %).^**1**^ **H NMR** (400 MHz; THF‐*d*_8_): *δ*=7.54 (t, ^3^ *J*(^1^H,^1^H)=7.8 Hz, 1H, *p*‐Dipp), 7.48 (t, ^3^ *J*(^1^H,^1^H)=7.8 Hz, 1H, *p*‐Dipp), 7.39 (d, ^3^ *J*(^1^H,^1^H)=7.8 Hz, 2H, *m*‐Dipp), 7.19 (d, ^3^ *J*(^1^H,^1^H)=7.8 Hz, 2H, *m*‐Dipp), 7.05 (s, 1H, HC=CB), 2.91 (sept, ^3^ *J*(^1^H,^1^H)=6.7 Hz, 2H, C*H*(CH_3_)_2_), 2.54 (sept, ^3^ *J*(^1^H,^1^H)=13.7, 6.8 Hz, 2H, C*H*(CH_3_)_2_), 1.26 (d, ^3^ *J*(^1^H,^1^H)=6.9 Hz, 6H, CH(C*H*_3_)_2_), 1.18 (d, ^3^ *J*(^1^H,^1^H)=6.8 Hz, 6H, CH(C*H*_3_)_2_), 1.12 (d, ^3^ *J*(^1^H,^1^H)=6.8 Hz, 6H, CH(C*H*_3_)_2_), 0.98 (d, ^3^ *J*(^1^H,^1^H)=6.6 Hz, 6H, CH(C*H*_3_)_2_) ppm. ^**11**^ **B NMR** (96 MHz; THF‐*d*_8_): *δ*=21.56 (br s, C_Carbene_‐B), −15.86 (s, HC=CB) ppm. ^**13**^ **C NMR** (126 MHz; THF‐*d*_8_): *δ*=153.41 (q, ^1^ *J*(^11^B,^13^C)=57.9 Hz, HC=*C*B), 149.91–149.73 (m, Ar^F^), 148.01–147.80 (m, Ar^F^), 145.66 (s, *o*‐Dipp), 144.43 (s, *o*‐Dipp), 140.01‐139.72 (m, Ar^F^), 137.99–137.52 (m, 2 ×Ar^F^), 135.78‐135.51 (m, Ar^F^), 134.80 (s, *ipso*‐Dipp) 134.24 (s, H*C*=CB), 134.12 (s, *ipso*‐Dipp), 130.35 (s, *p*‐Dipp), 130.27 (s, *p*‐Dipp), 124.13 (s, *m*‐Dipp), 123.60 (s, *m*‐Dipp), 28.38 (s, *C*H(CH_3_)_2_), 27.73 (s, *C*H(CH_3_)_2_), 24.73 (s, CH(*C*H_3_)_2_), 23.70 (s, CH(*C*H_3_)_2_), 23.03 (s, CH(*C*H_3_)_2_), 21.81 (s, CH(*C*H_3_)_2_) ppm. A signal corresponding to N−C−N was not observed. ^**19**^ **F NMR** (377 MHz; THF‐*d*_8_): *δ*=−126.96 − −131.28 (m, *o*‐Ar^F^), −162.43 (t, ^3^ *J*(^19^F,^19^F)=20.2 Hz, *p*‐Ar^F^), −167.48 (s, *m*‐Ar^F^) ppm. **EA** – Anal. calc. for C_45_H_35_B_2_Br_3_F_15_LiN_2_⋅C_4_H_10_O: C, 47.80; H, 3.68; N, 2.28. Found: C, 47.56; H, 4.29; N, 2.07.

[{(WCA‐IDipp)AlCl_3_}Li] (**3**). [(WCA‐IDipp)Li ⋅ toluene] (**1**, 2 g, 2 mmol, 1 eq.) was suspended in toluene (50 mL) and solid AlCl_3_ (267 mg, 2 mmol, 1 eq.) was added in one portion. The resulting pale brown suspension was stirred for 12 h at room temperature. Afterwards, the solvent was removed in vacuo, affording an off‐white solid. For purification, the solid was dissolved in THF (2 mL) and was layered with *n*‐hexane (6 mL) at ambient temperature. Crystals appeared within 3 d, which were collected by decantation of the supernatant solution. Residual solvent was removed in vacuo, yielding the product as a colorless solid (1.216 g, 0.968 mmol, 48 %). The bromide derivative can be obtained with the same procedure ^**1**^ **H NMR** (600 MHz; THF‐*d*_8_): *δ*=7.39 (t, ^3^ *J*(^1^H,^1^H)=7.8 Hz, 1H, *p*‐Dipp), 7.29 (t, ^3^ *J*(^1^H,^1^H)=7.8 Hz, 1H, *p*‐Dipp), 7.23 (d, ^3^ *J*(^1^H,^1^H)=7.8 Hz, 2H, *m*‐Dipp), 7.03 (d, ^3^ *J*(^1^H,^1^H)=7.6 Hz, 2H, *m*‐Dipp), 6.65 (s, 1H, HC=CB), 2.98 (sept, ^3^ *J*(^1^H,^1^H)=6.7 Hz, 2H, C*H*(CH_3_)_2_), 2.76 (sept, ^3^ *J*(^1^H,^1^H)=6.6 Hz, 2H, C*H*(CH_3_)_2_), 1.43 (d, ^3^ *J*(^1^H,^1^H)=6.7 Hz, 6H, CH(C*H*_3_)_2_), 1.29 (d, ^3^ *J*(^1^H,^1^H)=6.5 Hz, 6H, CH(C*H*_3_)_2_), 0.97 (d, ^3^ *J*(^1^H,^1^H)=6.7 Hz, 6H, CH(C*H*_3_)_2_), 0.93 (br s, 6H, CH(C*H*_3_)_2_) ppm. ^**11**^ **B NMR** (96 MHz; THF‐*d*_8_): *δ*=−15.69 (s, 1B) ppm. ^**13**^ **C NMR** (151 MHz; THF‐*d*_8_): *δ*=149.91 (q, ^1^ *J*(^11^B,^13^C)=62.7 Hz, HC=*C*B), 149.71–149‐53 (m, *ipso*‐Ar^F^), 148.11–141.93 (m, Ar^F^), 146.91 (s, *o*‐Dipp), 139.45–139.33 (m, Ar^F^), 137.80–137.65 (m, Ar^F^), 137.39–137.22 (m, Ar^F^), 135.75–135.59 (m, Ar^F^), 135.55 (s, *ipso*‐Dipp), 133.80 (s, *ipso*‐Dipp), 133.74 (br s, H*C*=CB), 130.02 (s, *p*‐Dipp), 129.41 (s, *p*‐Dipp), 123.32 (s, *m*‐Dipp), 122.99 (s, *m*‐Dipp), 28.59 (s, *C*H(CH_3_)_2_), 27.53 (s, *C*H(CH_3_)_2_), 26.62 (s, CH(*C*H_3_)_2_), 26.19 (s, CH(*C*H_3_)_2_), 22.95 (s, CH(*C*H_3_)_2_), 21.41 (s, CH(*C*H_3_)_2_) ppm. A signal corresponding to N−C−N was not observed. ^**19**^ **F NMR** (282 MHz; THF‐*d*_8_): *δ*=−128.50 − −133.00 (m, *o*‐Ar^F^), −163.70 (t, ^3^ *J*(^19^F,^19^F)=20.4 Hz, *p*‐Ar^F^), −167.87 (s, *m*‐Ar^F^) ppm. **EA** – Anal. calc. for C_45_H_35_BF_15_LiN_2_AlCl_3_ ⋅ 3(C_4_H_10_O): C, 54.24; H, 5.19; N, 2.22. Found: C, 53.78; H, 5.13; N, 1.85.

[{(WCA‐IDipp)GaCl_3_}Li] (**4**). GaCl_3_ (176 mg, 1 mmol, 1 eq.) was dissolved in toluene (40 mL) and [(WCA‐IDipp)Li ⋅ toluene] (**1**, 1 g, 1 mmol, 1 eq.) was added slowly as a solid in small portions. The resulting pale‐yellow cloudy solution was stirred for 2 h at room temperature and subsequently filtered over Celite, affording a yellow solution. The solvent was then removed in vacuo, and the resulting colorless solid, containing some oily residue, was washed with *n*‐hexane (3×5 mL). Residual solvent was removed in vacuo, yielding the product as a colorless solid (898 mg, 0.862 mmol, 86 %). ^**1**^ **H NMR** (600 MHz; THF‐*d*_8_): *δ*=7.39 (t, ^3^ *J*(^1^H,^1^H)=7.8 Hz, 1H, *p*‐Dipp), 7.30 (t, ^3^ *J*(^1^H,^1^H)=7.8 Hz, 1H, *p*‐Dipp), 7.24 (d, ^3^ *J*(^1^H,^1^H)=7.8 Hz, 2H *m*‐Dipp), 7.04 (d, ^3^ *J*(^1^H,^1^H)=7.7 Hz, 2H, *m*‐Dipp), 6.73 (s, 1H, HC=CB), 2.95 (sept, ^3^ *J*(^1^H,^1^H)=6.7 Hz, 2H, C*H*(CH_3_)_2_), 2.75 (sept, ^3^ *J*(^1^H,^1^H)=6.6 Hz, 2H, C*H*(CH_3_)_2_), 1.44 (d, ^3^ *J*(^1^H,^1^H)=6.7 Hz, 6H, CH(C*H*_3_)_2_), 1.28 (d, ^3^ *J*(^1^H,^1^H)=6.7 Hz, 6H, CH(C*H*_3_)_2_), 0.98 (d, ^3^ *J*(^1^H,^1^H)=6.8 Hz, 6H, CH(C*H*_3_)_2_), 0.93 (br s, 6H, CH(C*H*_3_)_2_) ppm. ^**11**^ **B NMR** (96 MHz; THF‐*d*_8_): *δ*=−15.69 (s, 1B) ppm. ^**13**^ **C NMR** (151 MHz; THF‐*d*_8_): *δ*=158.11 (s, N−C−N), 150.37 (q, ^1^ *J*(^11^B,^13^C)=61.6 Hz, HC=*C*B), 149.71–149.52 (m, Ar^F^), 148.12–147.98 (m, Ar^F^), 146.88 (s, *o*‐Dipp), 139.54‐139.35 (m, Ar^F^), 137.93–137.76 (m, Ar^F^), 137.45–137.34 (m, Ar^F^), 135.80–135.62 (m, Ar^F^), 134.66 (s, *ipso*‐Dipp), 133.82 (s, H*C*=CB), 133.20 (s, *ipso*‐Dipp), 130.19 (s, *p*‐Dipp), 129.66 (s, *p*‐Dipp), 123.48 (s, *m*‐Dipp), 123.05 (s, *m*‐Dipp), 28.66 (s, *C*H(CH_3_)_2_), 27.59 (s, *C*H(CH_3_)_2_), 26.56 (s, CH(*C*H_3_)_2_), 26.11 (s, CH(*C*H_3_)_2_), 22.88 (s, CH(*C*H_3_)_2_), 21.45 (s, CH(*C*H_3_)_2_) ppm. ^**19**^ **F NMR** (282 MHz; THF‐*d*_8_): *δ*=−127.09 − −132.64 (m, *o*‐Ar^F^), −163.51 (t, ^3^ *J*(^19^F,^19^F)=20.4 Hz, 3F, *p*‐Ar^F^), −167.76 (s, *m*‐Ar^F^) ppm. **EA** – Anal. calc. for C_45_H_35_BF_15_LiN_2_GaCl_3_: C, 49.93; H, 3.26; N, 2.59. Found C, 50.21; H 3.51; N, 2.27.

[{(WCA‐IDipp)InCl_3_}Li] (**5**). [(WCA‐IDipp)Li ⋅ toluene] (**1**, 500 mg, 0.5 mmol, 1 eq.) was dissolved in THF (4 mL) and solid InCl_3_ (111 mg, 0.5 mmol, 1 eq.) was added in one portion. The resulting mixture was stirred for 1 h at room temperature, and a clear colorless solution was obtained. The THF solution was then layered with *n*‐hexane (10 mL), and after storing overnight, colorless crystals had appeared, which were collected by decantation of the supernatant solution. Residual solvent was removed in vacuo, yielding the product as a colorless solid (434 mg, 0.400 mmol, 80 %). ^**1**^ **H NMR** (500 MHz; THF‐*d*_8_): *δ*=7.42 (t, ^3^ *J*(^1^H,^1^H)=7.8 Hz, 1H, *p*‐Dipp), 7.33 (t, ^3^ *J*(^1^H,^1^H)=7.8 Hz, 1H, *p*‐Dipp), 7.27 (d, ^3^ *J*(^1^H,^1^H)=7.8 Hz, 2H, *m*‐Dipp), 7.08 (d, ^3^ *J*(^1^H,^1^H)=7.8 Hz, 2H, *m*‐Dipp), 6.86 (s, 1H, HC=CB), 2.94 (t, ^3^ *J*(^1^H,^1^H)=6.7 Hz, 2H, C*H*(CH_3_)_2_), 2.72 (t, ^3^ *J*(^1^H,^1^H)=6.7 Hz, 2H, C*H*(CH_3_)_2_), 1.47 (d, ^3^ *J*(^1^H,^1^H)=6.7 Hz, 6H, CH(C*H*_3_)_2_), 1.26 (d, ^3^ *J*(^1^H,^1^H)=6.8 Hz, 6H, CH(C*H*_3_)_2_), 1.00 (d, ^3^ *J*(^1^H,^1^H)=6.7 Hz, 6H, CH(C*H*_3_)_2_), 0.94 (d, ^3^ *J*(^1^H,^1^H)=6.6 Hz, 6H, CH(C*H*_3_)_2_) ppm. ^**11**^ **B NMR** (96 MHz; THF‐*d*_8_): *δ*=−15.72 (s, 1B) ppm. ^**13**^ **C NMR** (126 MHz; THF‐*d*_8_): *δ*=164.74‐163.59 (m, N−C−N), 151.35 (q, ^1^ *J*(^11^B,^13^C)=61.1 Hz, HC=*C*B), 149.86–149.63 (m, Ar^F^), 147.93–147.72 (m, Ar^F^), 146.65 (s, *o*‐Dipp), 139.72‐139.54 (m, Ar^F^), 137.72–137.49 (m, 2×Ar^F^), 135.53–135.33 (m, Ar^F^), 134.57 (s, *ipso*‐Dipp), 134.06 (s, H*C*=CB), 133.00 (s, *ipso*‐Dipp), 130.51 (s, *p*‐Dipp), 130.14 (s, *p*‐Dipp), 123.91 (s, *m*‐Dipp 123.45 (s, *m*‐Dipp), 28.60 (s, *C*H(CH_3_)_2_), 27.56 (s, *C*H(CH_3_)_2_), 26.51 (s, CH(*C*H_3_)_2_), 26.33 (s, CH(*C*H_3_)_2_), 22.52 (s, CH(*C*H_3_)_2_), 21.43 (s, CH(*C*H_3_)_2_) ppm. ^**19**^ **F NMR** (282 MHz; THF‐*d*_8_): *δ*=−127.81 − −130.37 (m, *o*‐Ar^F^), −163.36 (t, ^3^ *J*(^19^F,^19^F)=20.3 Hz, 3F, *p*‐Ar^F^), −167.69 (s, *m*‐Ar^F^) ppm. **EA** – Anal. calc. for C_45_H_35_BF_15_LiN_2_InCl_3_ ⋅ 4(C_4_H_10_O): C, 51.74; H, 4.77; N, 1.98. Found: C, 51.51; H, 4.73; N, 1.83.

## Conflict of interest

The authors declare no conflict of interest.

## Supporting information

As a service to our authors and readers, this journal provides supporting information supplied by the authors. Such materials are peer reviewed and may be re‐organized for online delivery, but are not copy‐edited or typeset. Technical support issues arising from supporting information (other than missing files) should be addressed to the authors.

Supplementary
