# Supplementary material for: Group 13 Element Trihalide Complexes of Anionic N‐Heterocyclic Carbenes
Source: Chem Asian J. 2020 Feb 20;15(6):845–51. doi: 10.1002/asia.201901774 (PMC7154526; doi:10.1002/asia.201901774)
Supplement: Supplementary file 1 — Supplementary [file ASIA-15-845-s001.pdf]

CHEMISTRY
AN ASIAN JOURNAL

Supporting Information

Group 13 Element Trihalide Complexes of Anionic N-Heterocyclic Carbenes

Luong Phong Ho, Lisa Anders, and Matthias Tamm*© 2020 The Authors. Published by Wiley-VCH Verlag GmbH & Co. KGaA. This is an open access article under the terms of the Creative Commons Attribution License, which permits use, distribution and reproduction in any medium, provided the original work is properly cited.This manuscript is part of a special collection in honor of the 2nd International Conference on Organometallics and Catalysis (ICOC-2020). .

Content

S1 Crystallographic details 2
S1.1 (WCA-IDipp)$BBr_3Li(Et_2O)_2$ 3
S1.2 (WCA-IDipp)$AlCl_3Li$(toluene)$_2$ 6
S1.3 (WCA-IDipp)$AlBr_3Li$(toluene)$_2$ 9
S1.4 (WCA-IDipp)$GaCl_3$(benzene)$_2$ 12
S1.5 (WCA-IDipp)$InCl_3Li$(thf)$_4$ 15
S2 ^{1}H, ^{11}B, ^{13}C and ^{19}F NMR Spectra 17
S2.1 (WCA-IDipp)BBr_3Li 17
S2.2 (WCA-IDipp)$AlCl_3Li$(thf)$_3$ 21
S2.3 (WCA-IDipp)$GaCl_3Li$ 25
S2.4 (WCA-IDipp)$InCl_3Li$(thf)$_4$ 29
S3 References 33

S1 Crystallographic details

Suitable single crystals were mounted on a hair or on a MiTiGen mount in perfluorinated inert oil. The intensity measurements were performed at 100 K on an Oxford Diffraction Nova A and a Rigaku XtaLAB Synergy S Single Source diffractometer using mirror-focussed Cu*K*α radiation or on an Oxford Diffraction Eos using monochromated Mo*K*α radiation. The diffractometer software CrysAlisPRO was employed.[1] Absorption corrections were based on multiscans. The structures were refined anisotropically on F^2 using SHELXL-2017/1 or -2018/3.[2] Hydrogen atoms were included using a riding model or rigid methyl groups. Further details are given in Table S1-5.

Solvent content: The boron species **2** contains two coordinated diethylether molecules per asymmetric unit; both are ordered. The aluminum species **3** and **3A** both crystallize with two molecules of toluene per asymmetric unit. For **3**, both toluene molecules are ordered. For **3A**, the toluene molecule coordinated to the lithium atom in disordered over two positions; the other toluene molecule is ordered. The gallium species **4** crystallizes with two molecules of benzene per asymmetric unit; both are ordered. The indium species **5** crystallizes with four molecules of thf per asymmetric unit; these thf molecules are coordinated to the lithium ion and are all ordered.

Exceptions and special details: The disordered toluene molecule in **3A** was refined using appropriate restraints to improve stability of refinement, but the dimensions are not entirely satisfactory and should be interpreted with caution.

Complete data have been deposited with the Cambridge Crystallographic Data Centre under the CCDC numbers 1972068-1972072 for compounds **2**, **3**, **3A**, **4** and **5**. These data can be obtained free of charge from http://www.ccdc.cam.ac.uk/.

S1.1 (WCA-IDipp)$BBr_3Li(Et_2O)_2$

Table S1. Crystallographic data for compound **2**.

Compound	**2**	
Identification code	1972068	
Empirical formula	$C_{53}H_{55}B_2Br_3F_{15}LiN_2O_2$	
Formula weight	1305.28	
Temperature	100(2) K	
Wavelength	1.54184 Å	
Instrument (scan mode)	Xcalibur, Atlas, Nova (ω scan)	
Crystal system	Triclinic	
Space group	*P*-1	
Unit cell dimensions	a = 10.3432(4) Å	α = 73.340(4)°
	b = 14.2625(6) Å	β = 79.620(3)°
	c = 19.6159(8) Å	γ = 78.645(4)°
Volume	2694.3(2) $Å^3$	
Z	2	
Density (calculated)	1.609 Mg/m^3	
Absorption coefficient	3.616 mm^{-1}	
F(000)	1312	
Crystal habitus	irregular (colorless)	
Crystal size	0.177 x 0.116 x 0.066 mm^3	
Theta range for data collection	3.507 to 77.469°	
Index ranges	-12<=h<=12, -17<=k<=17, -24<=l<=24	
Reflections collected	25161	
Independent reflections	25161 [R(int) = NA]	
Completeness to theta = 67.684°	99.8 %	
Absorption correction	Gaussian	
Max. and min. transmission	1.000 and 0.880	
Refinement method	Full-matrix least-squares on F^2	
Data / restraints / parameters	25161 / 0 / 716	
Goodness-of-fit on F^2	0.958	
Final R indices [I>2sigma(I)]	R1 = 0.0647, wR2 = 0.1710	
R indices (all data)	R1 = 0.0933, wR2 = 0.1860	
Largest diff. peak and hole	2.503 and -1.782 e.$Å^{-3}$	
Crystallisation Details:	A saturated solution of WCA-IDipp-BBr_3Li in Et_2O was stored at -40°C for several days.	
Solution:	SHELXT-2014/5 (Sheldrick 2015)	
Refinement:	SHELXL-2018/3 (Sheldrick 2018)	
Interface:	OLEX2 v1.2	
Measurement and Refinement Details:	Refined as a 2-component twin.	

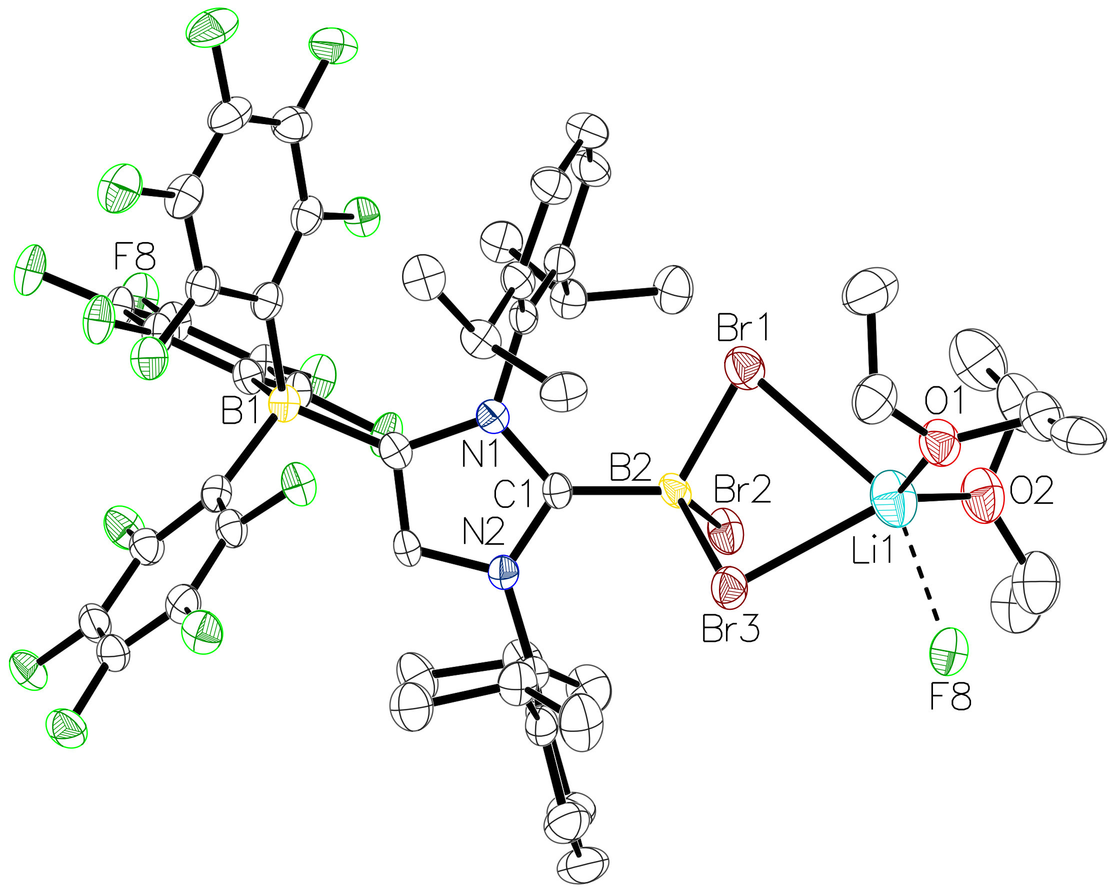

Figure S1. Molecular structure of **2**·$2Et_2O$ with thermal displacement parameters drawn at 50% probability; hydrogen atoms are omitted for clarity.

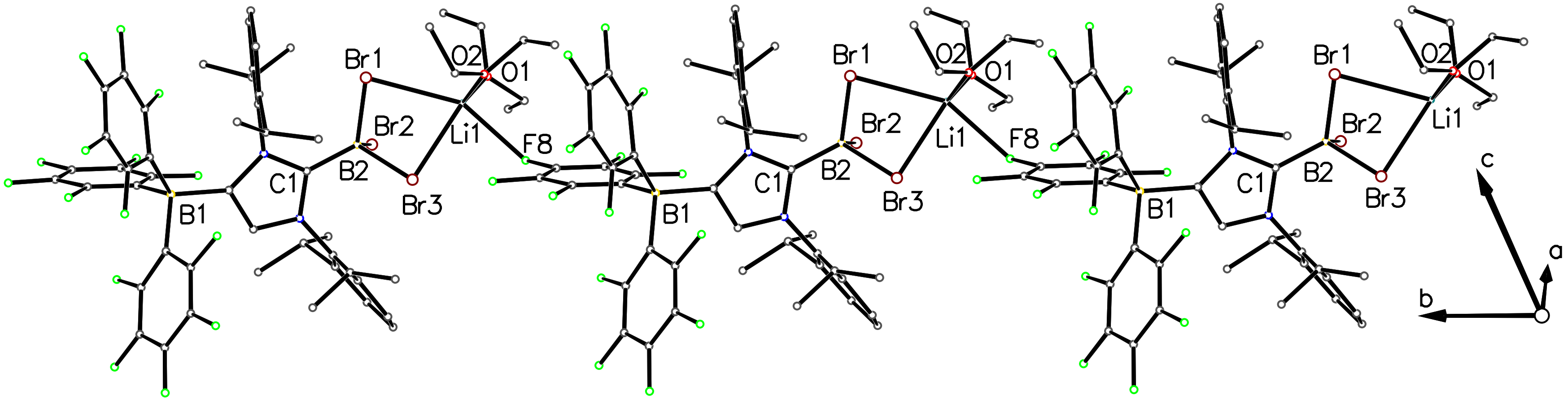

Figure S2. Chain polymer of **2**·2Et_2O. Hydrogen atoms are omitted for clarity. The overall chain direction is parallel to the b axis.

S1.2 (WCA-IDipp)$AlCl_3Li$(toluene)$_2$

Table S2. Crystallographic data for compound **3**.

Compound	**3**	
Identification code	1972069	
Empirical formula	$C_{59}H_{51}AlBCl_3F_{15}LiN_2$	
Formula weight	1224.09	
Temperature	100(2) K	
Wavelength	1.54184 Å	
Instrument (scan mode)	Xcalibur, Atlas, Nova (ω scan)	
Crystal system	Monoclinic	
Space group	$P2_1/c$	
Unit cell dimensions	a = 10.70510(10) Å	α = 90°
	b = 24.6975(2) Å	β = 101.2920(10)°
	c = 21.6231(2) Å	γ = 90°
Volume	5606.25(9) Å^3	
Z	4	
Density (calculated)	1.450 Mg/m^3	
Absorption coefficient	2.436 mm^{-1}	
F(000)	2504	
Crystal habitus	needle (colorless)	
Crystal size	0.234 x 0.043 x 0.034 mm^3	
Theta range for data collection	3.579 to 76.233°	
Index ranges	-13<=h<=12, -31<=k<=31, -27<=l<=27	
Reflections collected	115766	
Independent reflections	11696 [R(int) = 0.0788]	
Completeness to theta = 67.684°	100.0 %	
Absorption correction	Gaussian	
Max. and min. transmission	0.994 and 0.972	
Refinement method	Full-matrix least-squares on F^2	
Data / restraints / parameters	11696 / 12 / 749	
Goodness-of-fit on F^2	1.027	
Final R indices [I>2sigma(I)]	R1 = 0.0423, wR2 = 0.1077	
R indices (all data)	R1 = 0.0556, wR2 = 0.1162	
Largest diff. peak and hole	0.853 and -0.772 e.Å^{-3}	
Crystallisation Details:	A saturated solution of WCA-IDipp-$AlCl_3Li$ in toluene was layered with hexane at ambient temperatures.	
Solution:	SHELXT-2014/5 (Sheldrick 2015)	
Refinement:	SHELXL-2017/1 (Sheldrick 2017)	
Interface:	WinGX v2014.1	

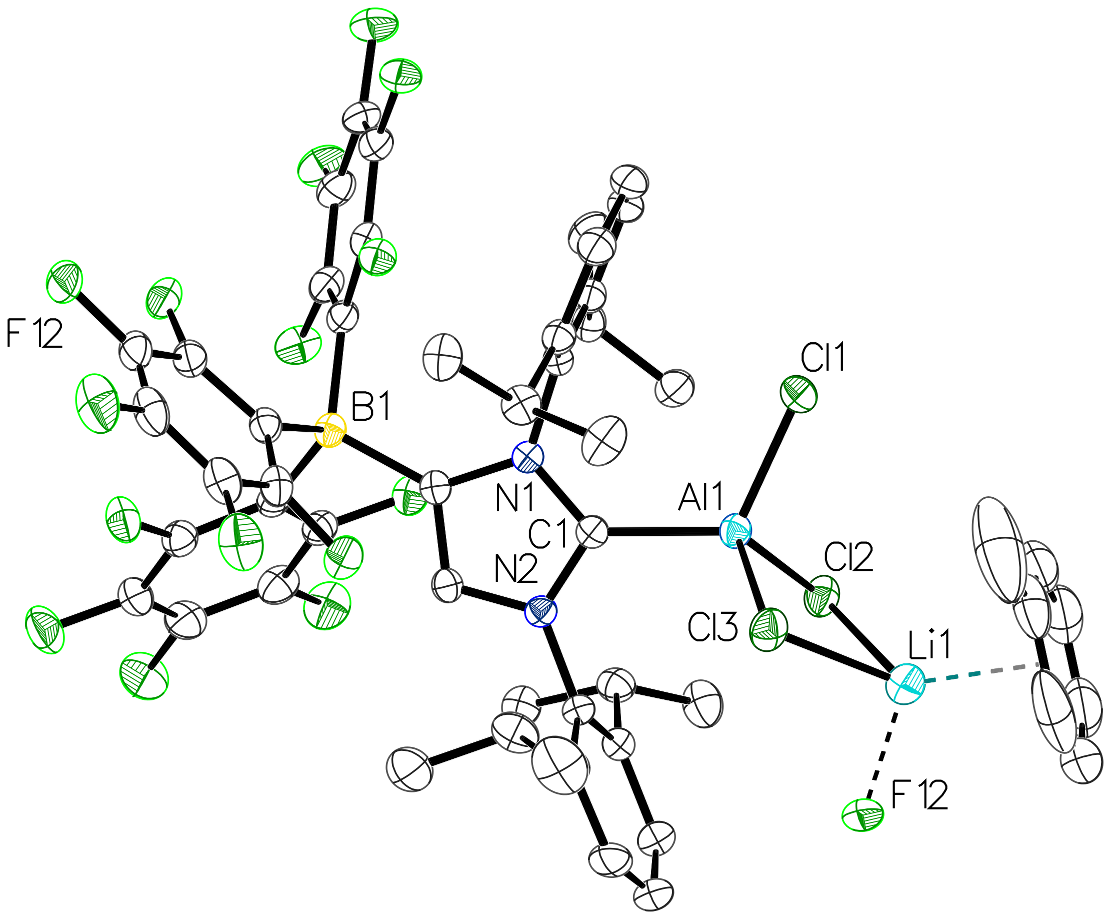

Figure S3. Molecular structure of **3**·2(toluene) with thermal displacement parameters drawn at 50% probability; hydrogen atoms and the second toluene molecule are omitted for clarity.

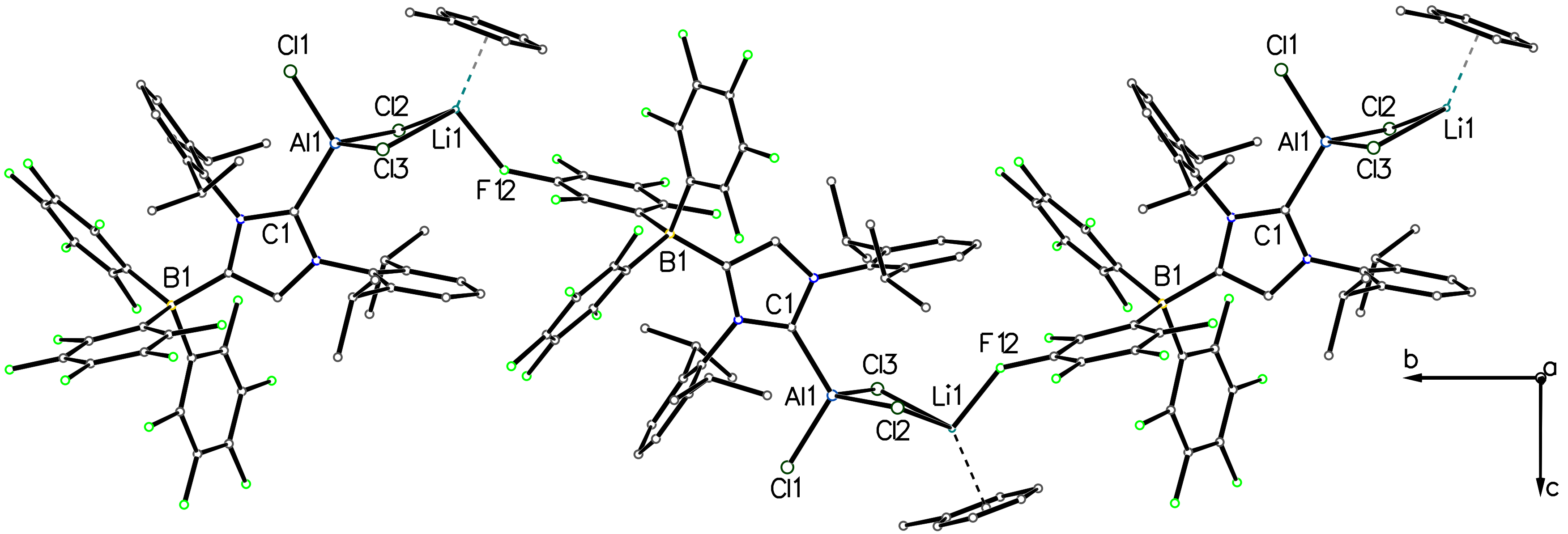

Figure S4. Chain polymer of **3**·2(toluene). Hydrogen atoms and the second toluene molecule are omitted for clarity. The overall chain direction is parallel to the b axis and the view direction is along the a axis.

S1.3 (WCA-IDipp)$AlBr_3Li(toluene)_2$

Table S3. Crystallographic data for compound 3A.

Compound	**3A**	
Identification code	1972070	
Empirical formula	$C_{59}H_{51}AlBBr_3F_{15}LiN_2$	
Formula weight	1357.47	
Temperature	293(2) K	
Wavelength	0.71073 Å	
Instrument (scan mode)	Xcalibur, Eos (ω scan)	
Crystal system	Monoclinic	
Space group	$P2_1/c$	
Unit cell dimensions	a = 10.73080(10) Å	α = 90°
	b = 24.8510(2) Å	β = 101.6960(10)°
	c = 21.7187(2) Å	γ = 90°
Volume	5671.49(9) Å^3	
Z	4	
Density (calculated)	1.590 Mg/m^3	
Absorption coefficient	2.240 mm^{-1}	
F(000)	2720	
Crystal habitus	clear block	
Crystal size	0.197 x 0.241 x 0.545 mm^3	
Theta range for data collection	2.143 to 29.130°	
Index ranges	-14<=h<=14, -34<=k<=34, -29<=l<=29	
Reflections collected	464775	
Independent reflections	15254 [R(int) = 0.0602]	
Completeness to theta = 25.242°	99.9 %	
Absorption correction	Analytical	
Max. and min. transmission	0.965 and 0.472	
Refinement method	Full-matrix least-squares on F^2	
Data / restraints / parameters	15254 / 99 / 814	
Goodness-of-fit on F^2	1.030	
Final R indices [I>2sigma(I)]	R1 = 0.0404, wR2 = 0.1100	
R indices (all data)	R1 = 0.0537, wR2 = 0.1179	
Largest diff. peak and hole	1.384 and -2.116 e.Å^{-3}	
Crystallisation Details:	A saturated solution of WCA-IDipp-$AlBr_3Li$ in toluene was layered with hexane at ambient temperatures.	
Solution:	SHELXT-2014/5 (Sheldrick 2015)	
Refinement:	SHELXL-2017/1 (Sheldrick 2017)	
Interface:	OLEX2 v1.2	
Measurement and Refinement Details:	The toluene molecule which is coordinated to the lithium atom is disordered and was refined over two positions.	

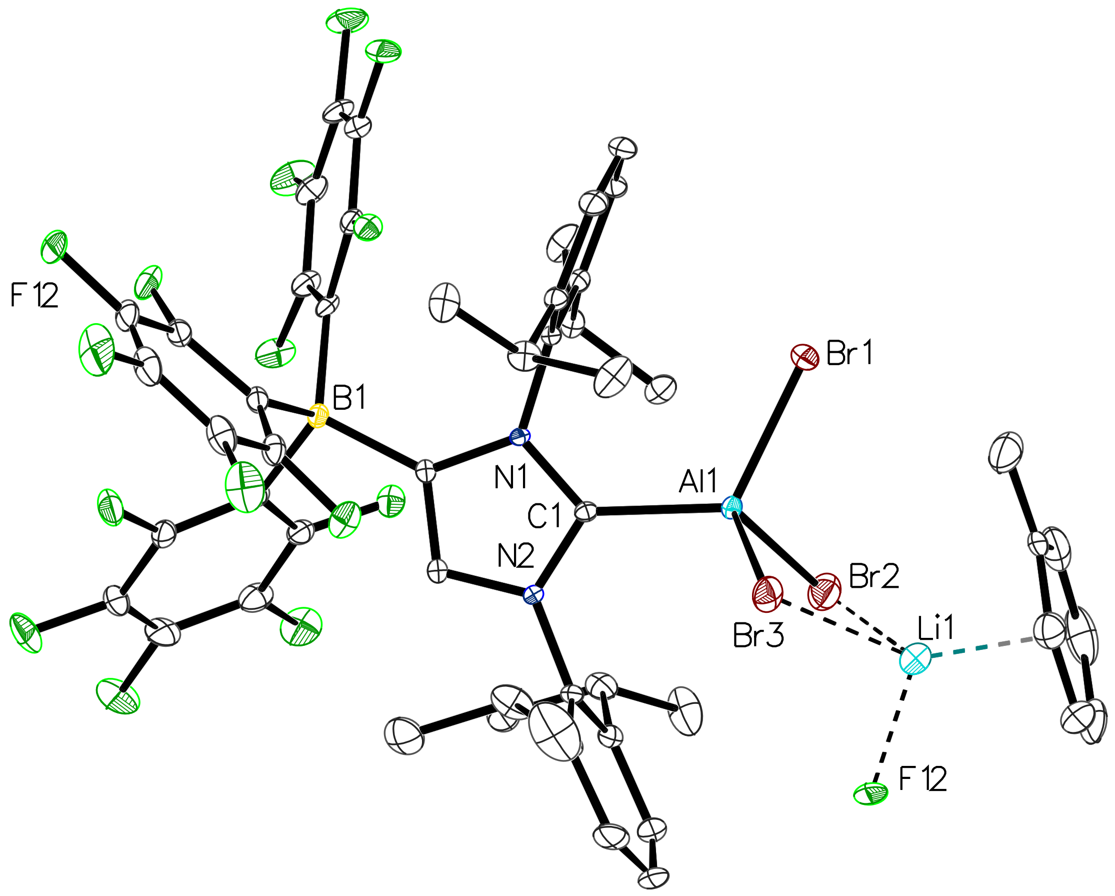

Figure S5. Molecular structure of **3A**·2(toluene) with thermal displacement parameters drawn at 50% probability; hydrogen atoms, the second position of the coordinated toluene molecule depicted and the second toluene molecule are omitted for clarity. Selected bond lengths [Å] and angles [°]: C1-Al1 2.019(2), Al1-Br1 2.2737(8), Al1-Br2 2.3034(9), Al1-Br3 2.3265(8), N1-C1-N2 104.68(18).

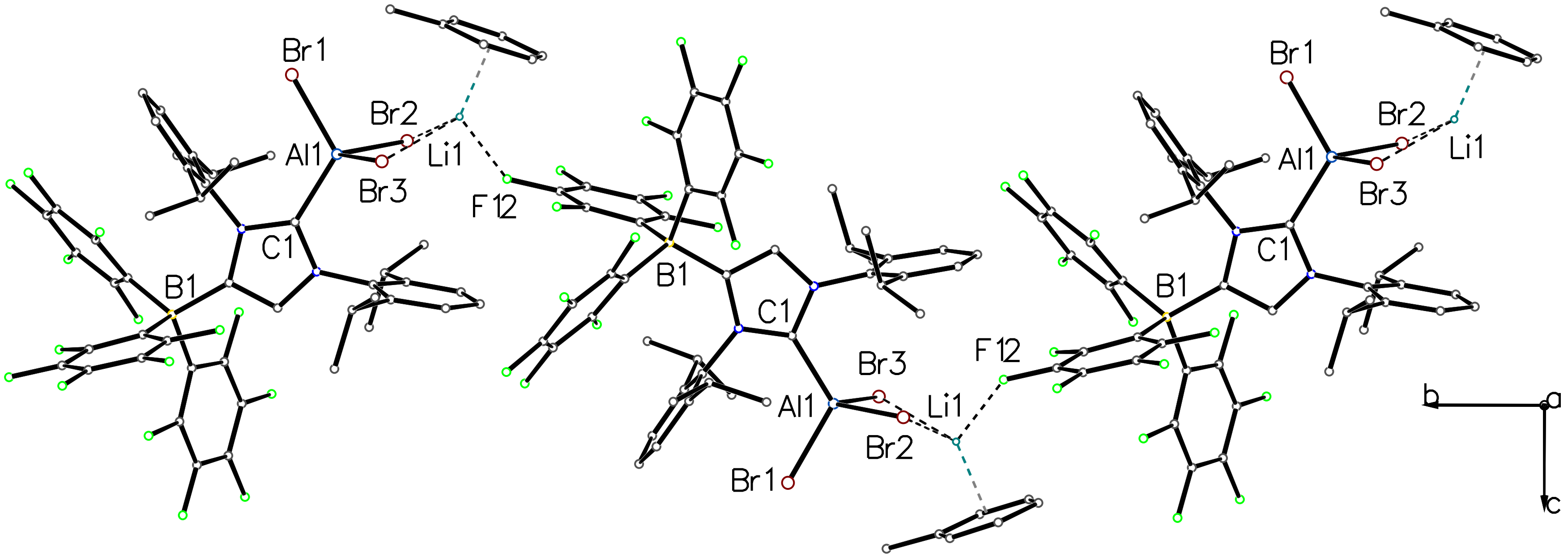

Figure S6. Chain polymer of **3A**·2(toluene). Hydrogen atoms, the second position of the coordinated toluene molecule depicted and the second toluene molecule are omitted for clarity. The overall chain direction is parallel to the b axis and the view direction is along the a axis.

S1.4 (WCA-IDipp)$GaCl_3$(benzene)$_2$

Table S4. Crystallographic data for compound 4.

Compound	**4**	
Identification code	1972071	
Empirical formula	$C_{57}H_{47}BCl_3F_{15}GaLiN_2$	
Formula weight	1238.78	
Temperature	100(2) K	
Wavelength	1.54184 Å	
Instrument (scan mode)	Xcalibur, Atlas, Nova (ω scan)	
Crystal system	Monoclinic	
Space group	$P2_1/c$	
Unit cell dimensions	a = 10.88710(10) Å	α = 90°
	b = 20.5088(2) Å	β = 94.8310(10)°
	c = 25.0378(2) Å	γ = 90°
Volume	5570.61(9) $Å^3$	
Z	4	
Density (calculated)	1.477 Mg/m^3	
Absorption coefficient	2.795 mm^{-1}	
F(000)	2512	
Crystal habitus	block (clear colourless)	
Crystal size	0.269 x 0.209 x 0.188 mm^3	
Theta range for data collection	3.543 to 76.420°	
Index ranges	-12<=h<=13, -25<=k<=25, -31<=l<=31	
Reflections collected	113618	
Independent reflections	11619 [R(int) = 0.0595]	
Completeness to theta = 67.684°	100.0 %	
Refinement method	Full-matrix least-squares on F^2	
Data / restraints / parameters	11619 / 46 / 729	
Goodness-of-fit on F^2	1.036	
Final R indices [I>2sigma(I)]	R1 = 0.0465, wR2 = 0.1262	
R indices (all data)	R1 = 0.0559, wR2 = 0.1348	
Largest diff. peak and hole	0.817 and -0.764 e.$Å^{-3}$	
Crystallisation Details:	A saturated solution of WCA-IDipp-$GaCl_3$Li in C_6D_6 was stored under inert atmosphere at ambient temperatures	
Solution:	SHELXT-2014/5 (Sheldrick 2015)	
Refinement:	SHELXL-2017/1 (Sheldrick 2017)	
Interface:	WinGX v2014.1	

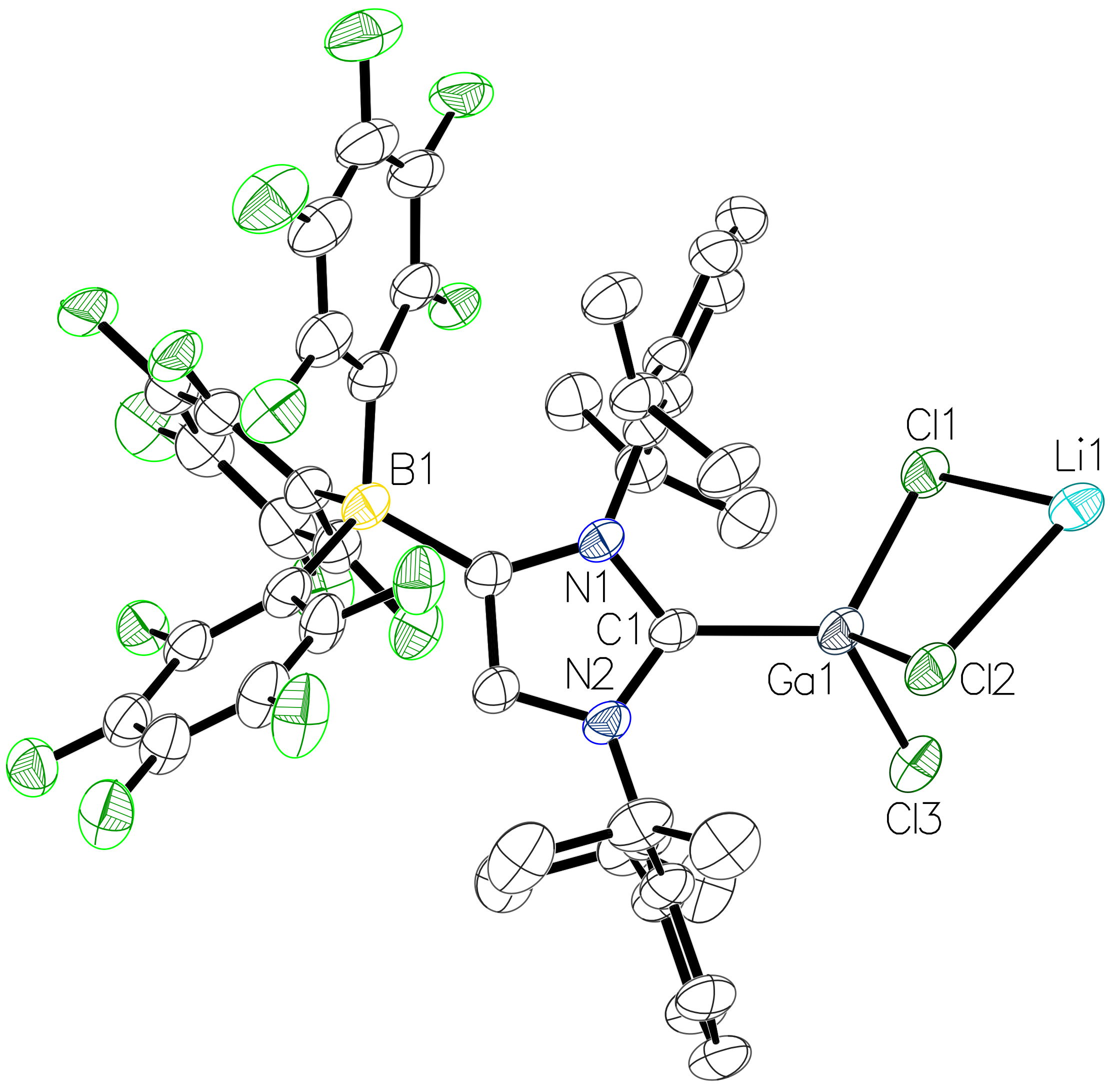

Figure S7. Molecular structure of **4**·benzene with thermal displacement parameters drawn at 50% probability; hydrogen atoms and one benzene molecule are omitted for clarity.

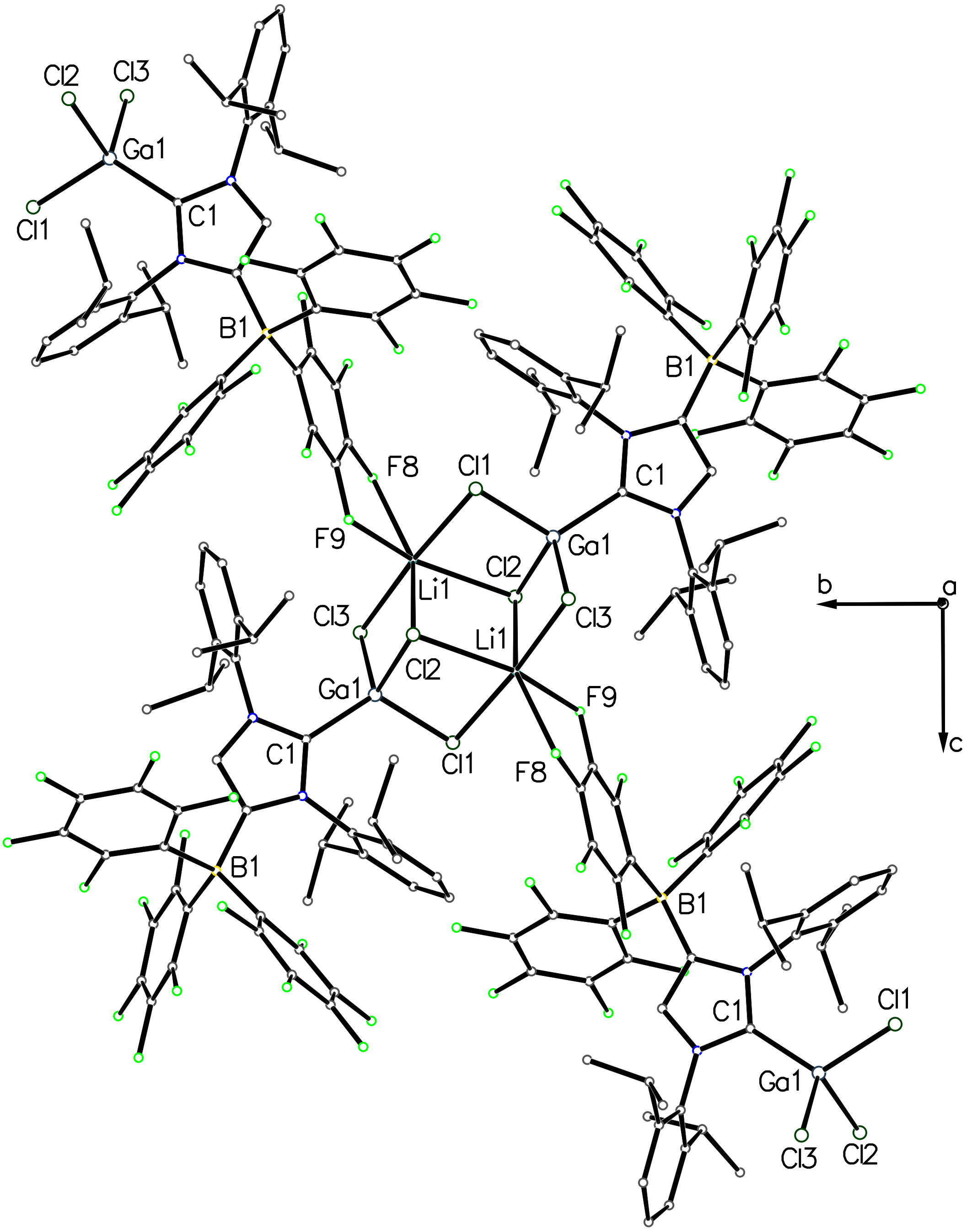

Figure S8. Polymer of **4**·benzene. Hydrogen atoms and the benzene molecule are omitted for clarity. The overall polymer expansion is within the bc plane and the view direction is along the a axis.

S1.5 (WCA-IDipp)$InCl_3Li(thf)_4$

Table S5. Crystallographic data for compound 5.

Compound	**4**	
Identification code	1972072	
Empirical formula	$C_{61}H_{67}BCl_3F_{15}InLiN_2O_4$	
Formula weight	1416.08	
Temperature	100(2) K	
Wavelength	1.5418 Å	
Instrument (scan mode)	Xcalibur, Atlas, Nova (ω scan)	
Crystal system	Monoclinic	
Space group	$P2_1/c$	
Unit cell dimensions	a = 10.7080(2) Å	α= 90°
	b = 18.4890(4) Å	β= 95.130(2)°
	c = 31.7640(6) Å	γ = 90°
Volume	6263.5(2) $Å^3$	
Z	4	
Density (calculated)	1.502 Mg/m^3	
Absorption coefficient	5.002 mm^{-1}	
F(000)	2888	
Crystal habitus	irregular (clear colourless)	
Crystal size	0.770 x 0.720 x 0.296 mm^3	
Theta range for data collection	3.677 to 76.406°	
Index ranges	-13<=h<=11, -23<=k<=23, -39<=l<=40	
Reflections collected	126801	
Independent reflections	13062 [R(int) = 0.0627]	
Completeness to theta = 67.680°	100.0 %	
Absorption correction	Gaussian	
Max. and min. transmission	1.000 and 0.713	
Refinement method	Full-matrix least-squares on F^2	
Data / restraints / parameters	13062 / 60 / 801	
Goodness-of-fit on F^2	1.027	
Final R indices [I>2sigma(I)]	R1 = 0.0592, wR2 = 0.1594	
R indices (all data)	R1 = 0.0644, wR2 = 0.1644	
Largest diff. peak and hole	2.072 and -1.501 e.$Å^{-3}$	
Crystallisation Details:	A saturated solution of WCA-IDipp-$InCl_3Li$ in THF was layered with hexane at ambient temperatures.	
Solution:	SHELXT 2018/2 (Sheldrick 2018)	
Refinement:	SHELXL-2018/3 (Sheldrick 2018)	
Interface:	OLEX2 v1.2	

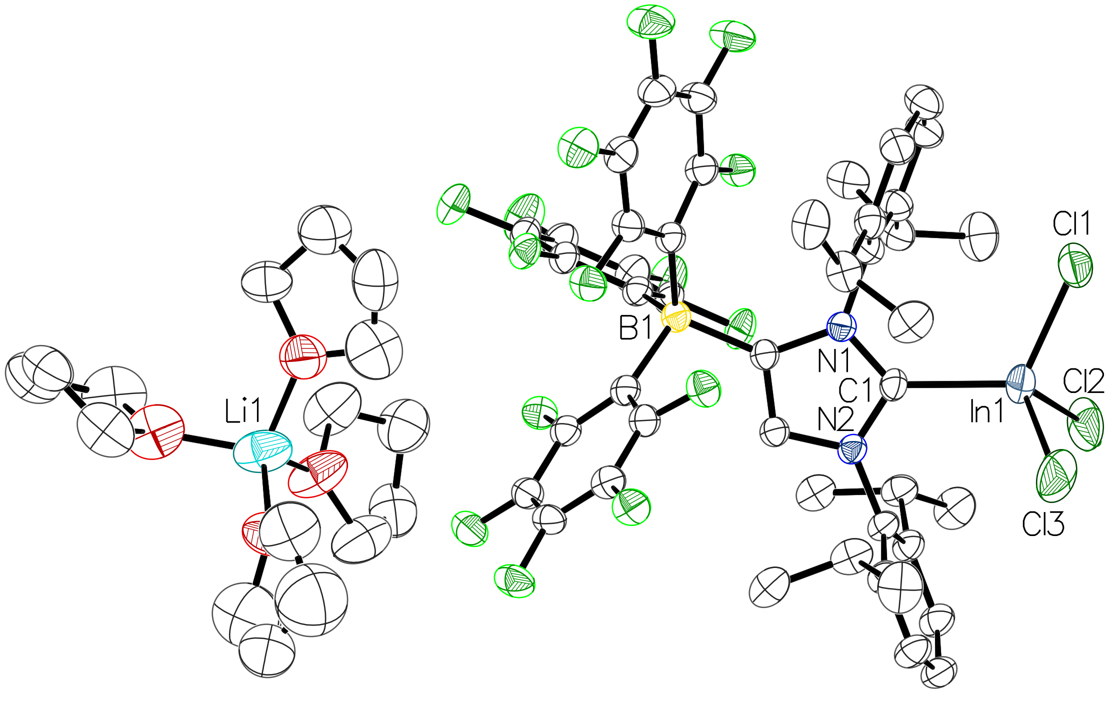

Figure S9. Molecular structure of **5**·4(thf) with thermal displacement parameters drawn at 50% probability; hydrogen atoms are omitted for clarity.

S2 ^{1}H, ^{11}B, ^{13}C and ^{19}F NMR Spectra

S2.1 (WCA-IDipp)BBr_3Li

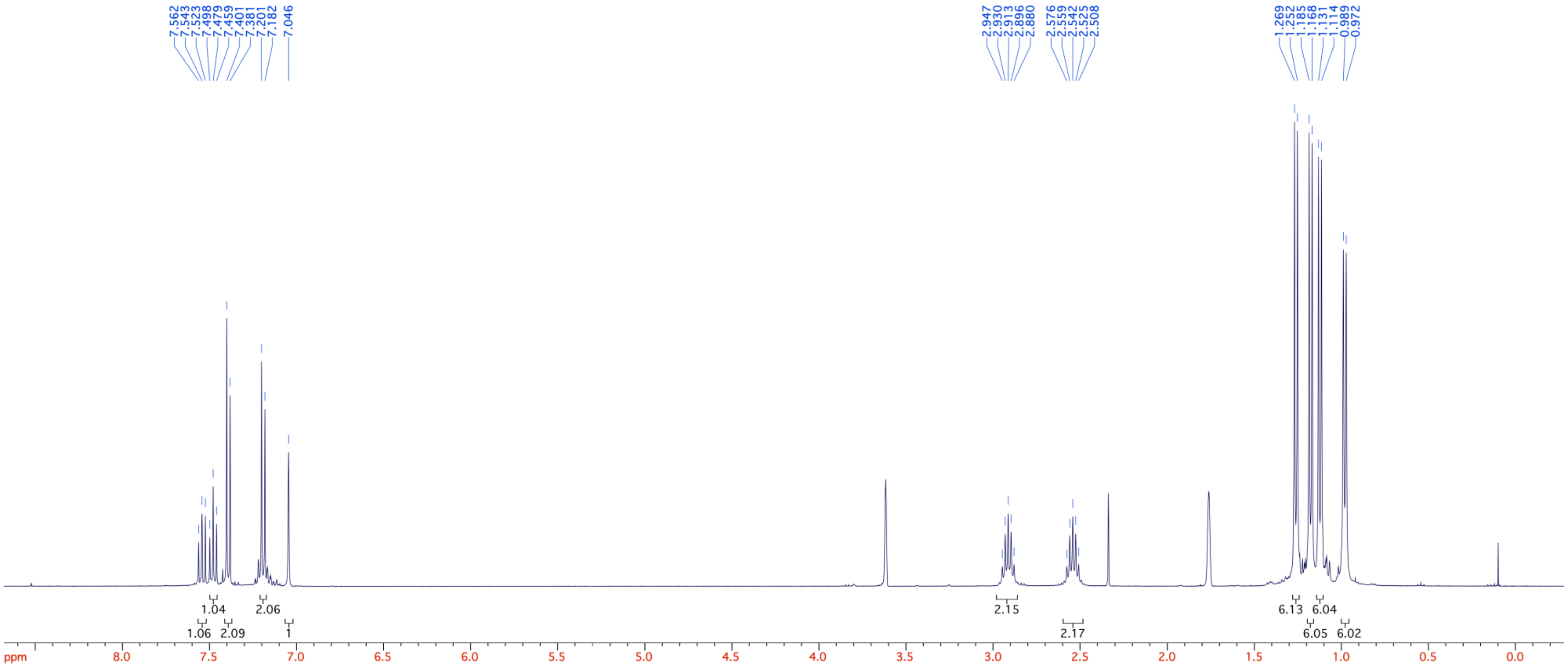

Figure S10. ^{1}H NMR spectrum (400 MHz, THF-d_8, 298K) of (WCA-IDipp)BBr_3Li (**2**).

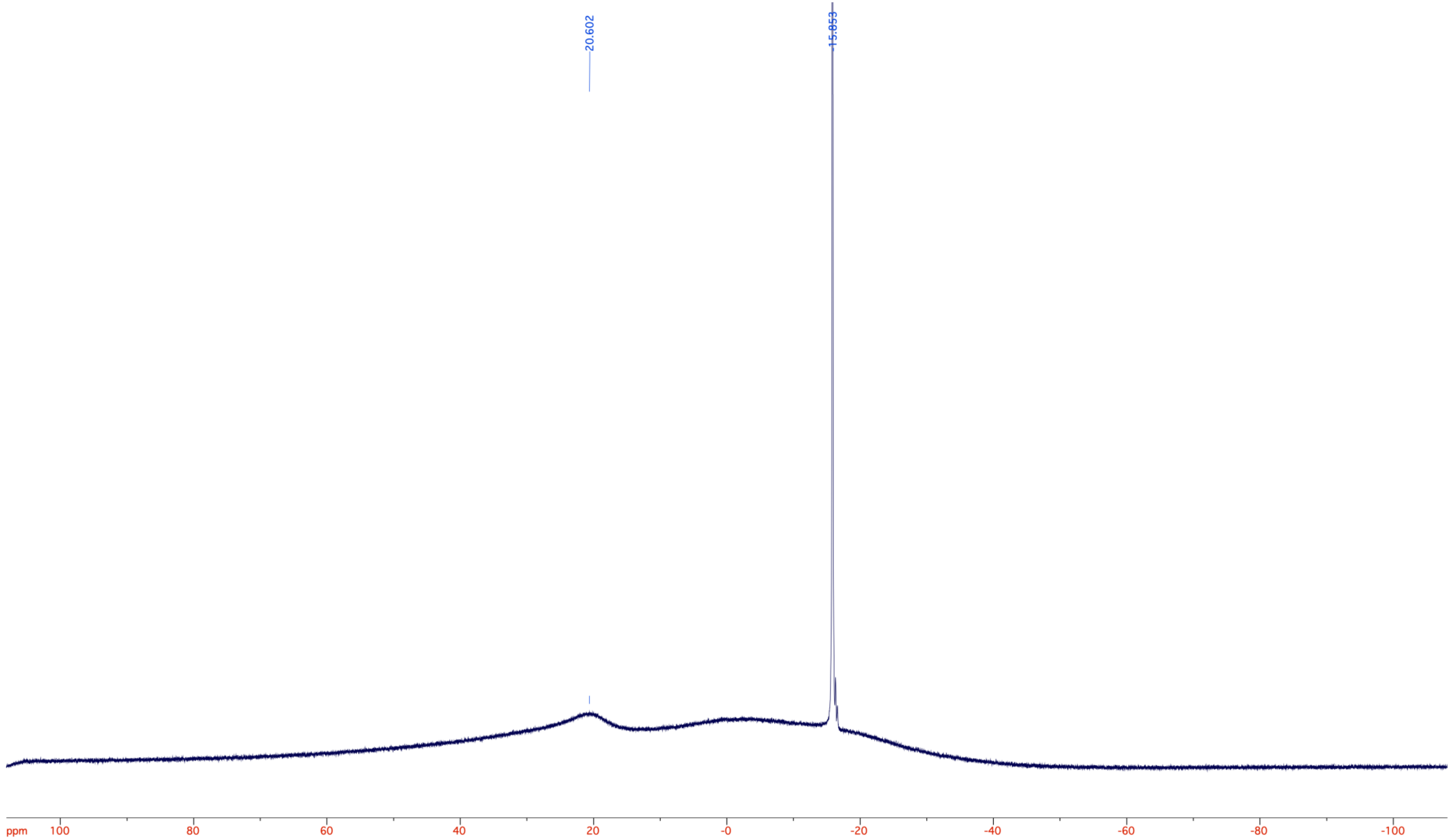

Figure S11. ^{11}B NMR spectrum (128 MHz, THF-d_8, 298K) of (WCA-IDipp)BBr_3Li (**2**).

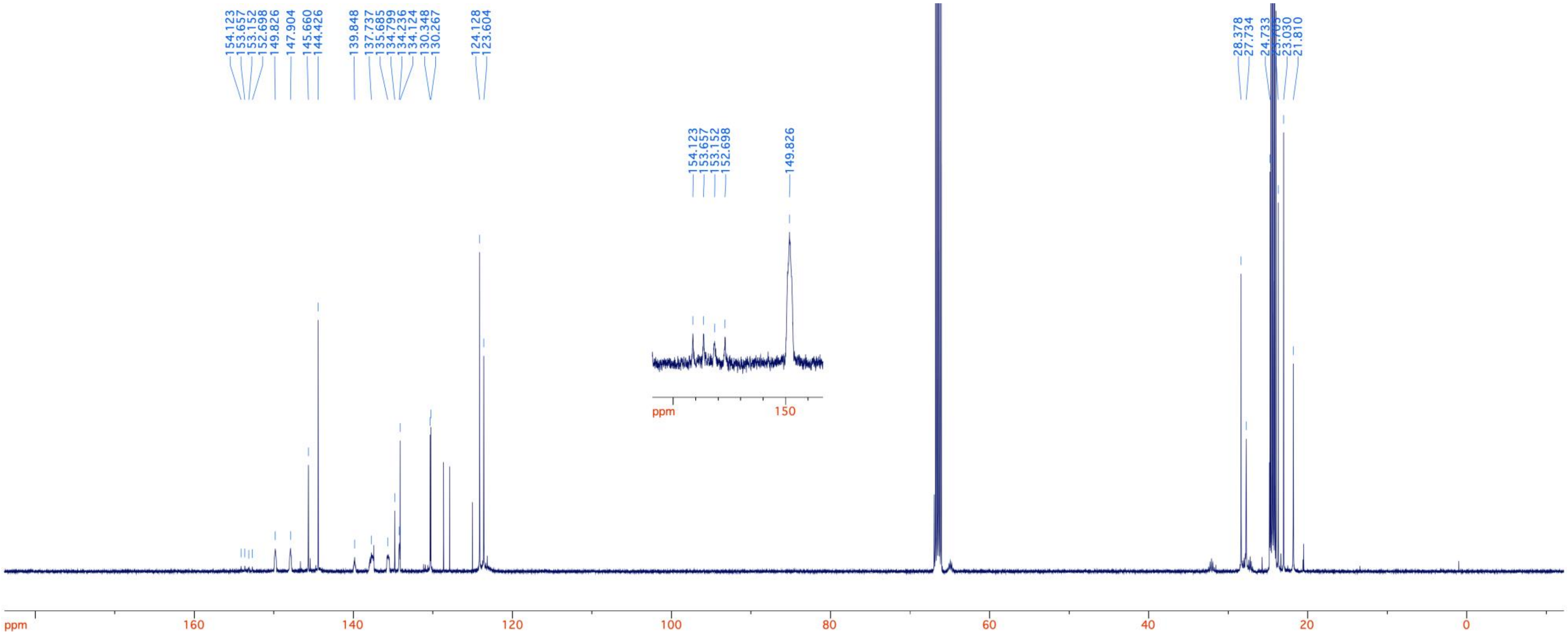

Figure S12. ^{13}C NMR spectrum (126 MHz, THF-d_8, 298K) of (WCA-IDipp)BBr_3Li (**2**).

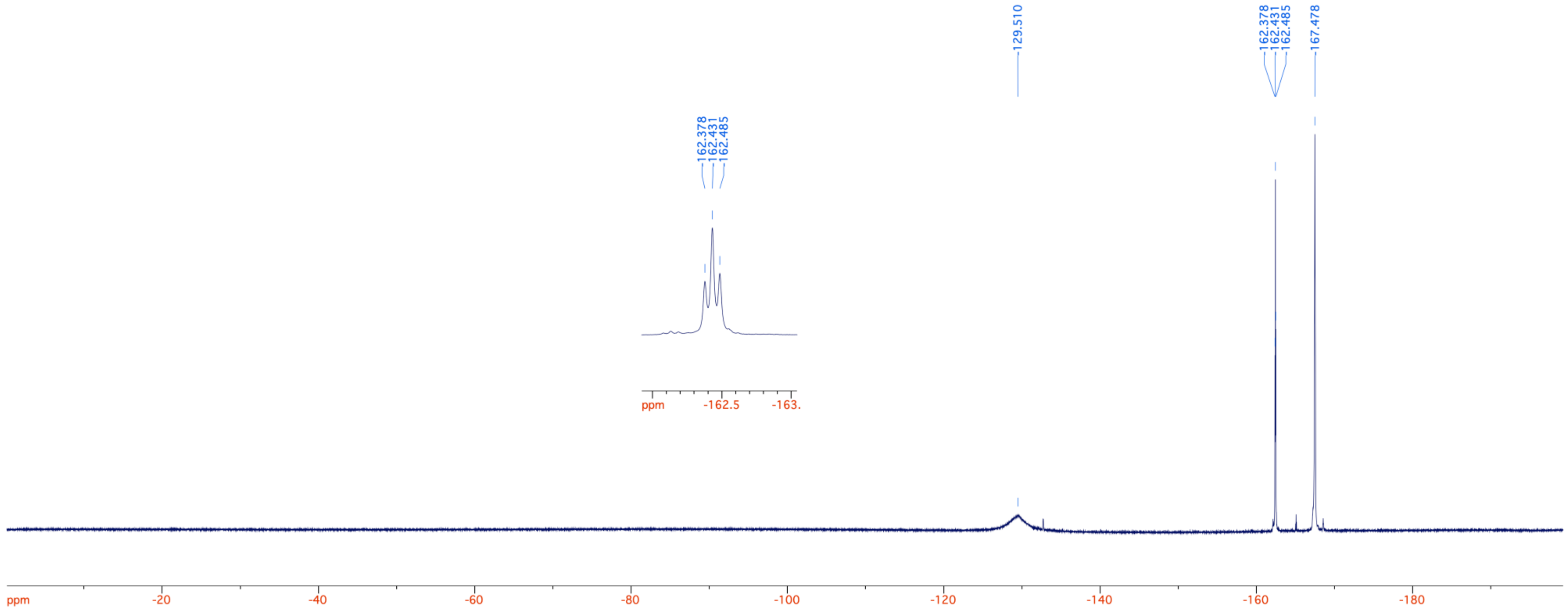

Figure S13. ^{19}F NMR spectrum (377 MHz, THF-d_8, 298K) of (WCA-IDipp)BBr_3Li (**2**).

S2.2 (WCA-IDipp)$AlCl_3Li(thf)_3$

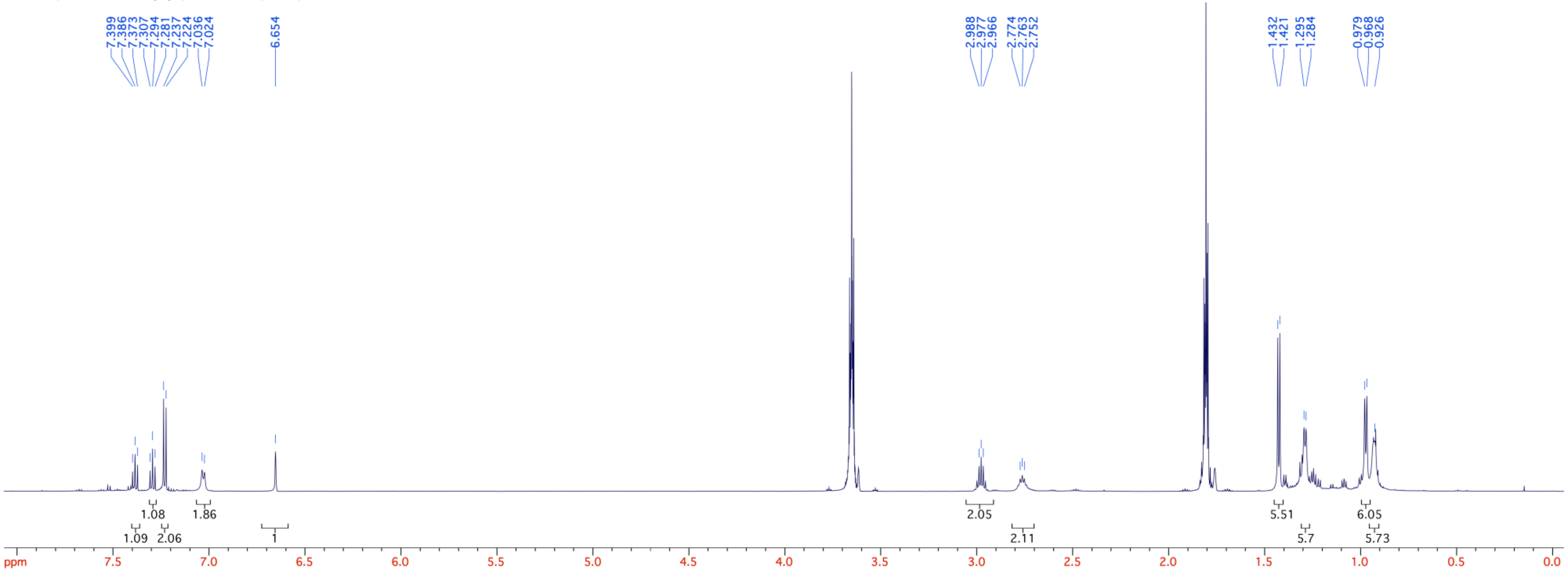

Figure S14. ^1H NMR spectrum (600 MHz, THF-d_8, 298K) of (WCA-IDipp)$AlCl_3Li(thf)_3$ (**3**·3(thf)).

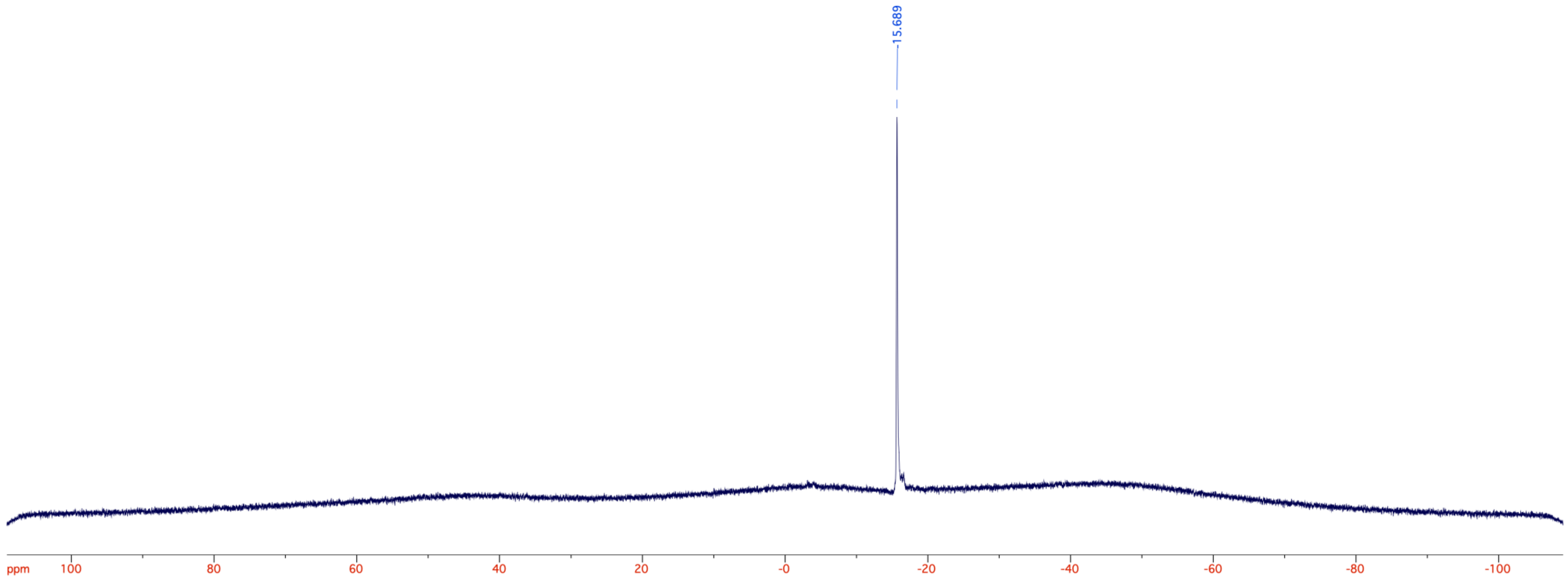

Figure S15. ^{11}B NMR spectrum (96 MHz, THF-d_8, 298K) of (WCA-IDipp)$AlCl_3Li(thf)_3$ (**3**·3(thf)).

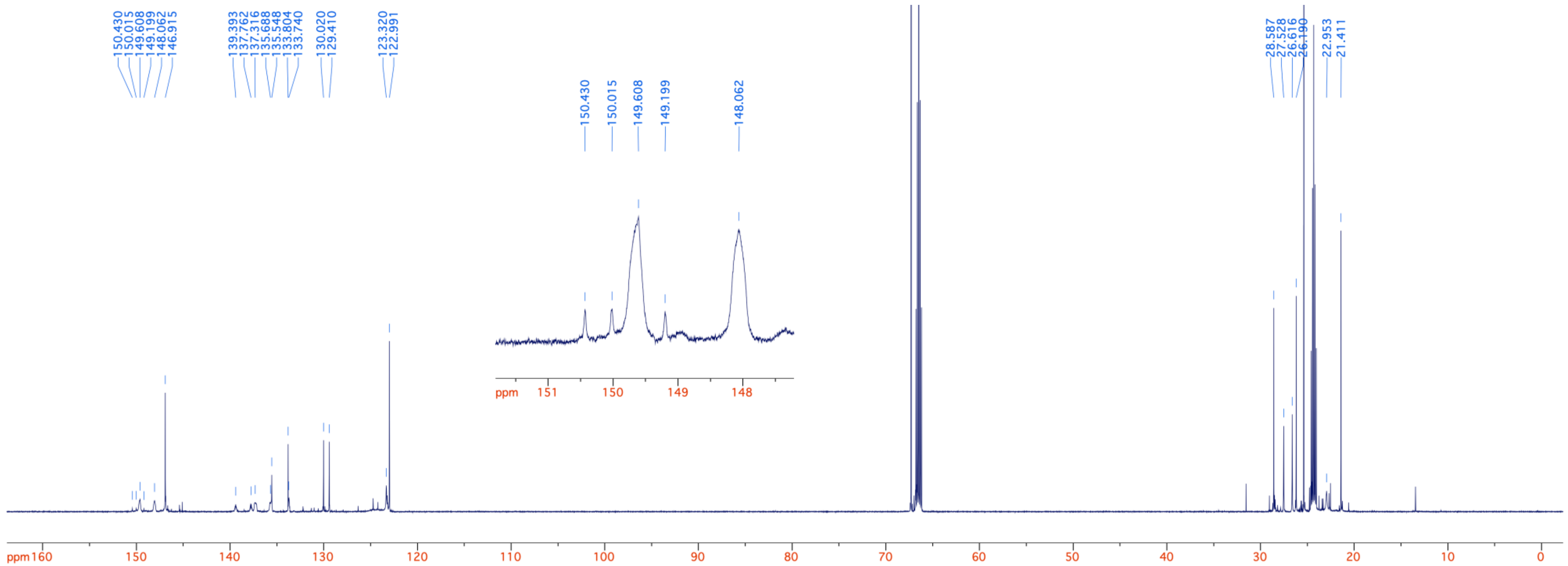

Figure S16. ^{13}C NMR spectrum (151 MHz, THF-d_8, 298K) of (WCA-IDipp)$AlCl_3Li(thf)_3$ (**3**·3(thf)).

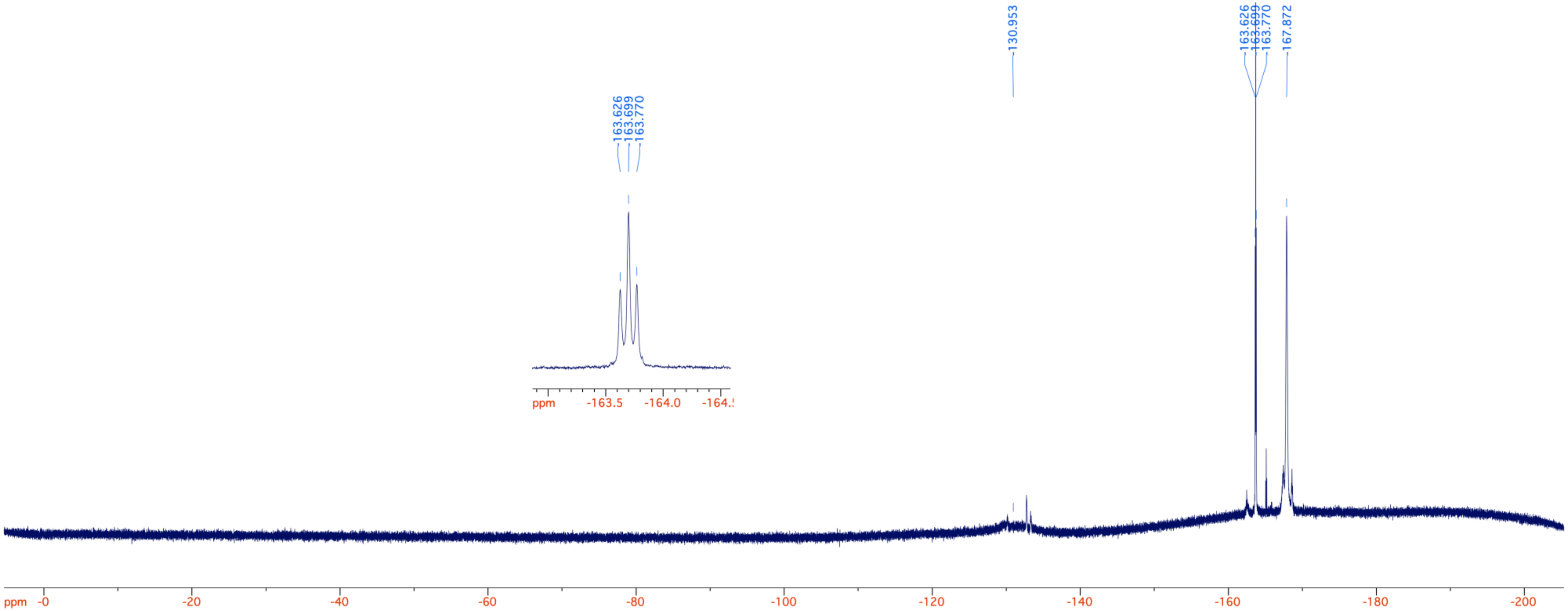

Figure S17. ^{19}F NMR spectrum (282 MHz, THF-d_8, 298K) of (WCA-IDipp)$AlCl_3Li(thf)_3$ (**3**·3(thf)).

S2.3 (WCA-IDipp)$GaCl_3Li$

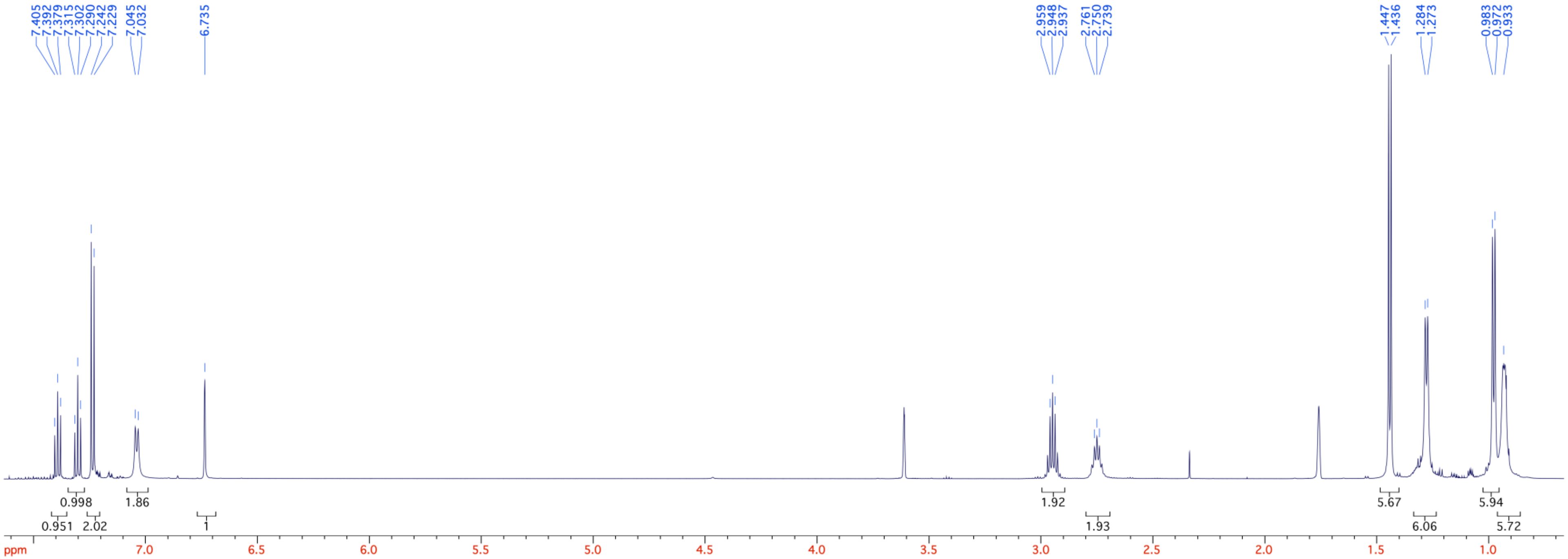

Figure S18. ^1H NMR spectrum (600 MHz, THF-d_8, 298K) of (WCA-IDipp)$GaCl_3Li$ (**4**).

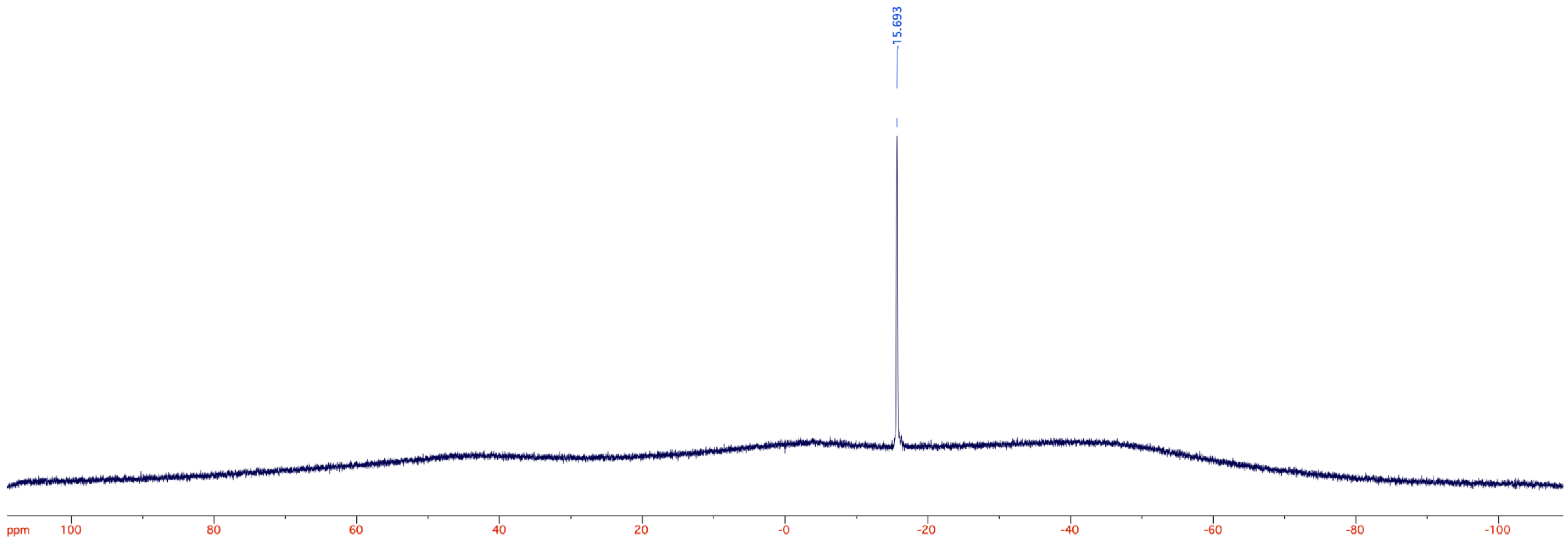

Figure S19. ^{11}B NMR spectrum (96 MHz, THF-d_8, 298K) of (WCA-IDipp)$GaCl_3Li$ (**4**).

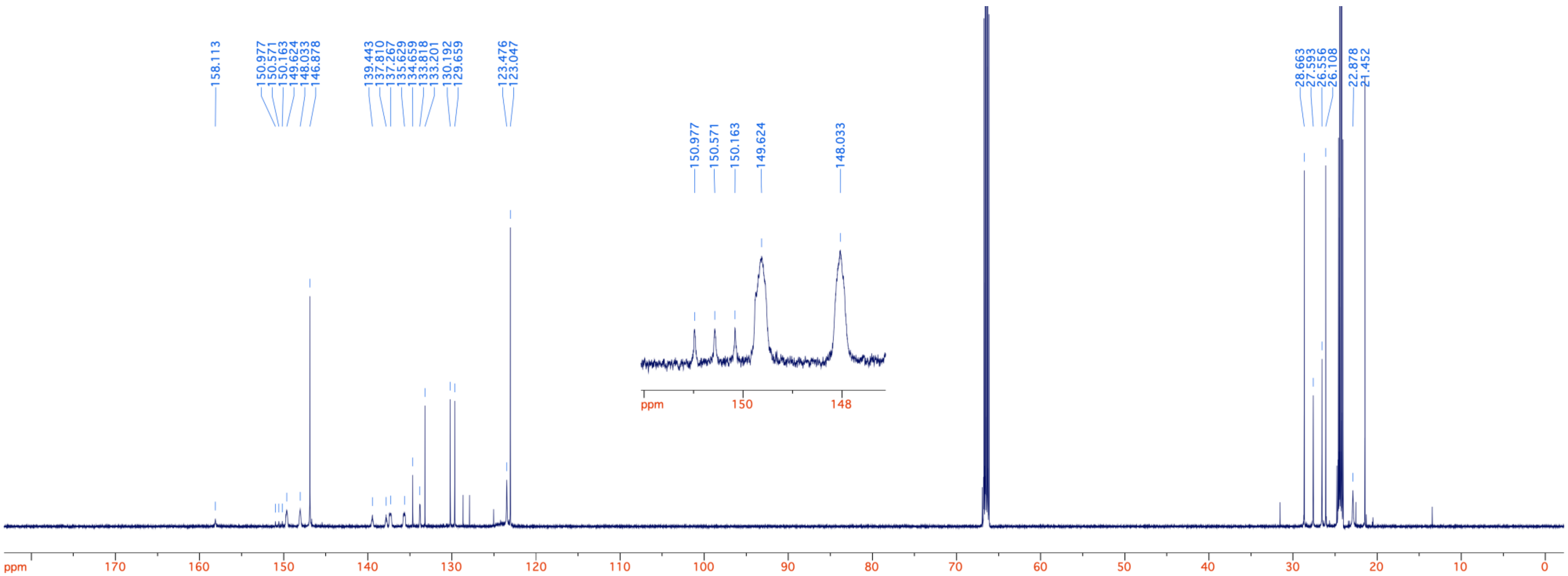

Figure S20. ^{13}C NMR spectrum (151 MHz, THF-d_8, 298K) of (WCA-IDipp)$GaCl_3Li$ (**4**).

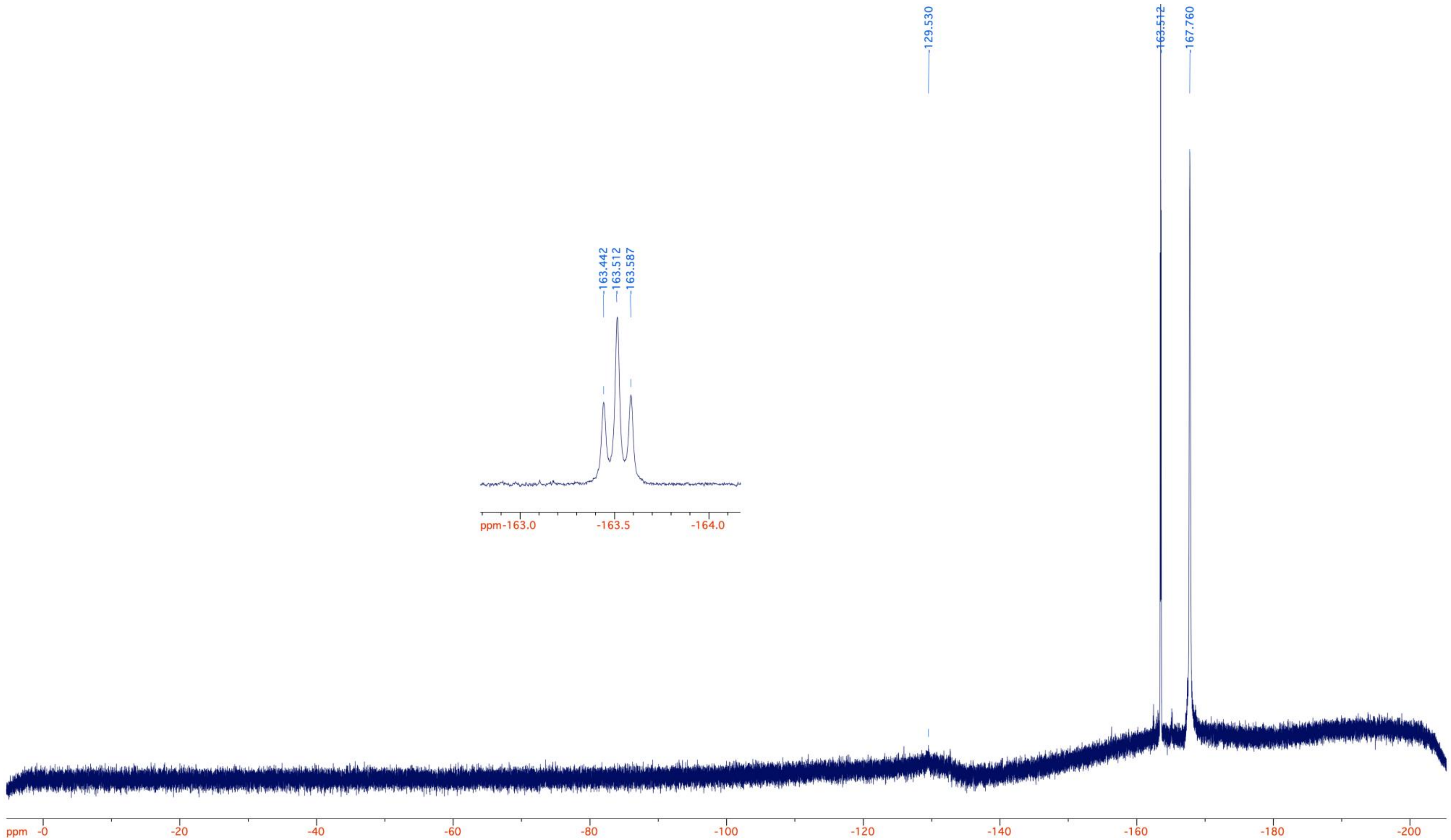

Figure S21. ^{19}F NMR spectrum (282 MHz, THF-d_8, 298K) of (WCA-IDipp)$GaCl_3Li$ (**4**).

S2.4 (WCA-IDipp)$InCl_3Li(thf)_4$

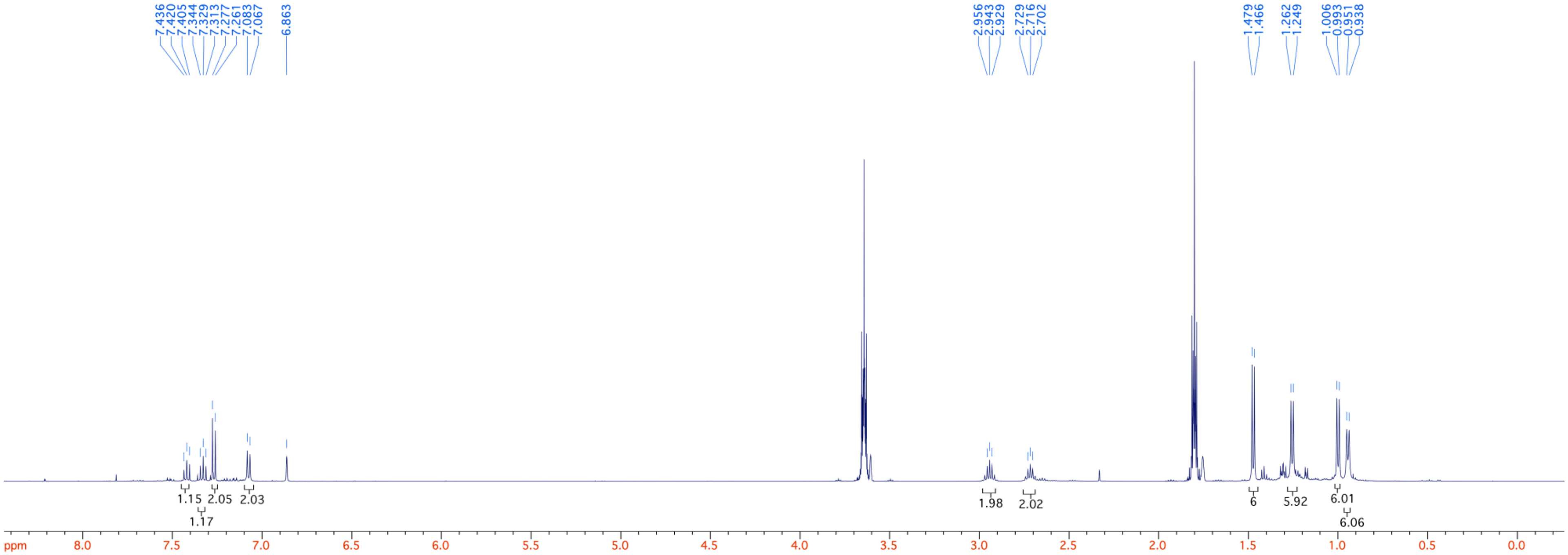

Figure S22. ^{1}H NMR spectrum (500 MHz, THF-d_8, 298K) of (WCA-IDipp)$InCl_3Li(thf)_4$ (**5**·4(thf)).

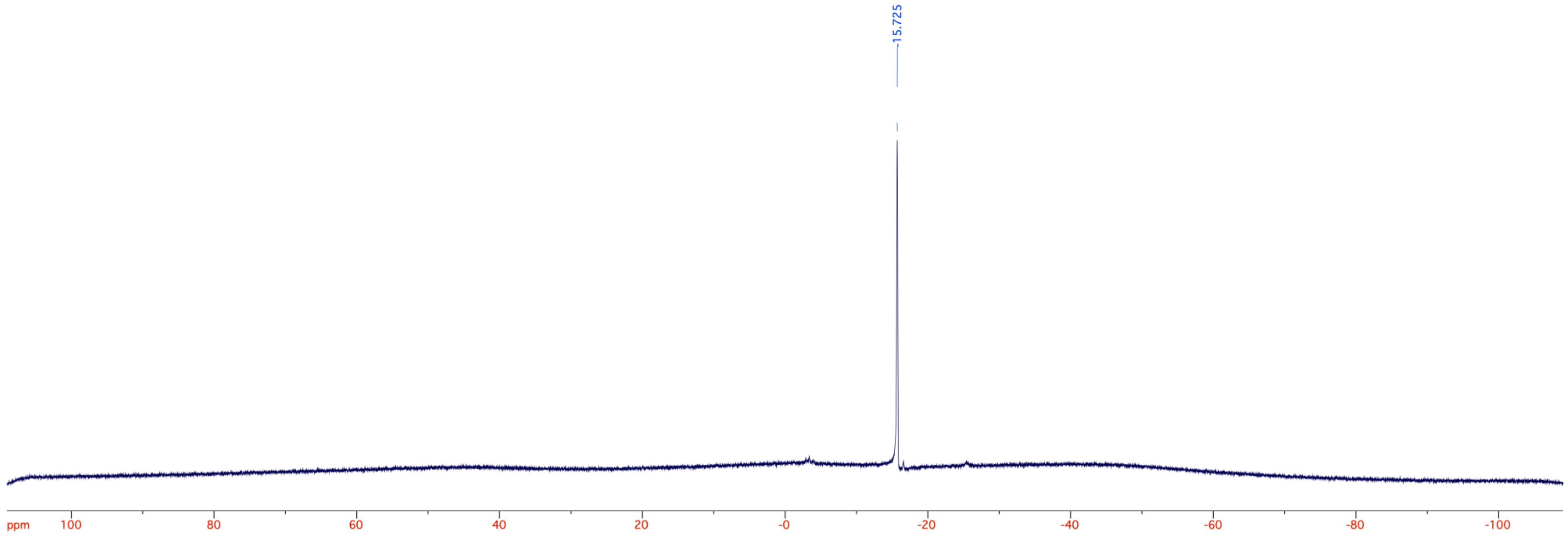

Figure S23. ^{11}B NMR spectrum (96 MHz, THF-d_8, 298K) of (WCA-IDipp)$InCl_3Li(thf)_4$ (**5**·4(thf)).

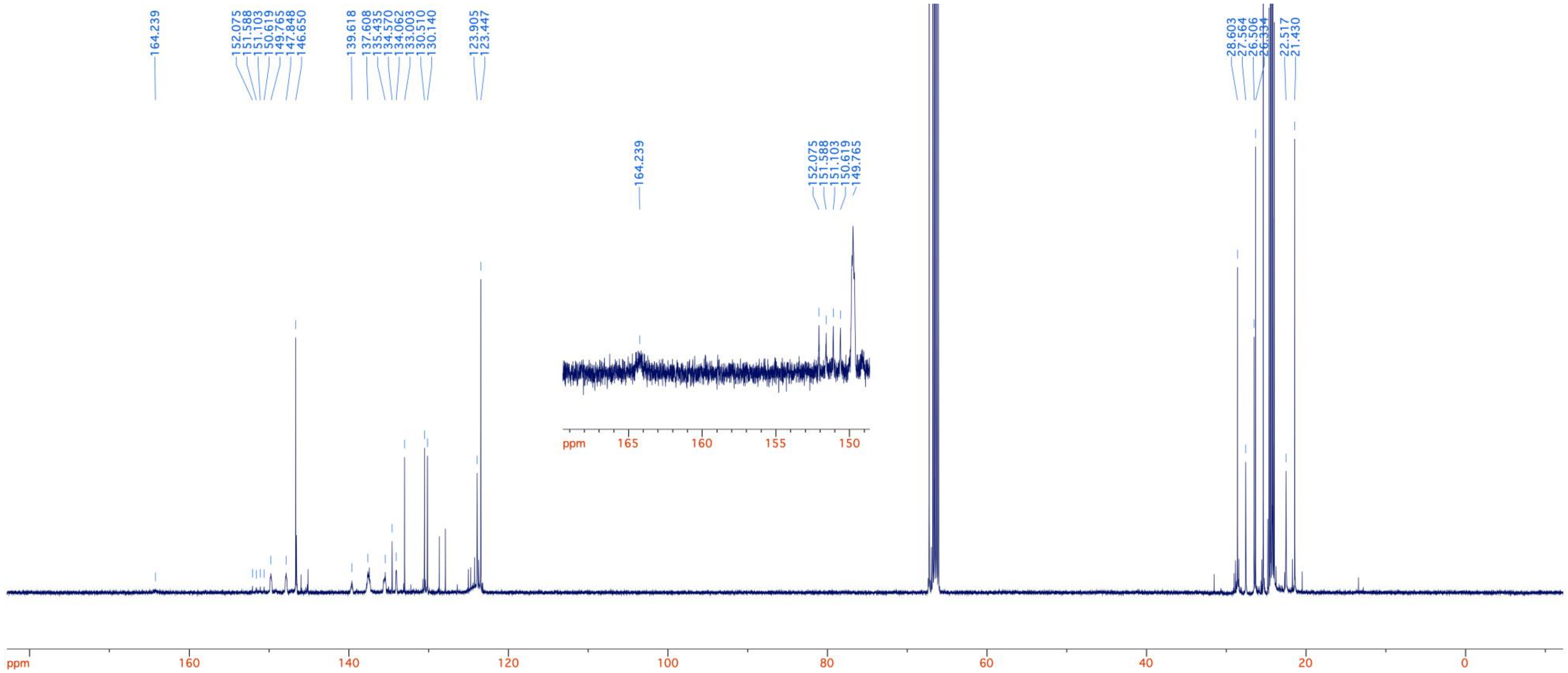

Figure S24. ^{13}C NMR spectrum (126 MHz, THF-d_8, 298K) of (WCA-IDipp)InCl$_3$Li(thf)$_4$ (**5**·4(thf)).

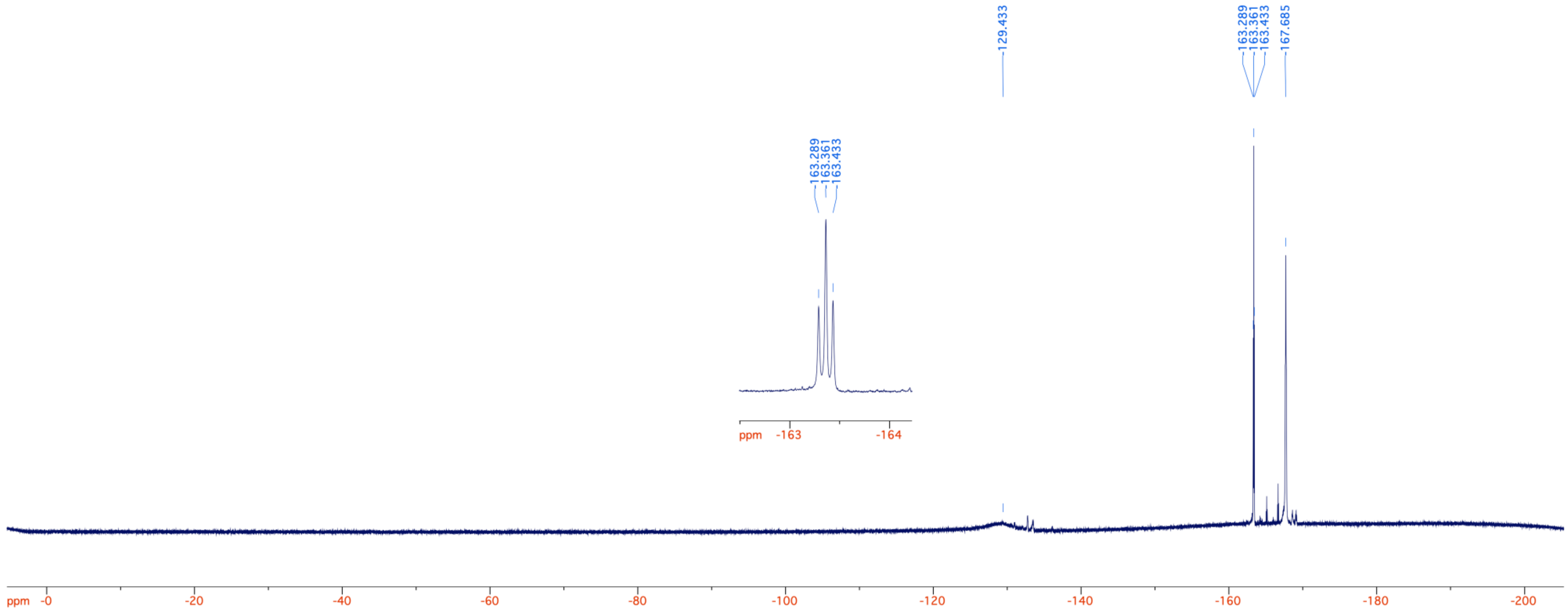

Figure S25. ^{19}F NMR spectrum (282 MHz, THF-d_8, 298K) of (WCA-IDipp)InCl$_3$Li(thf)$_4$ (**5**·4(thf)).

S3 References

[1] CrysAlisPRO, Oxford Diffraction/Agilent Technologies UK Ltd, Yarnton, England.
[2] Sheldrick, G. M. *Acta Crystallogr., Sect. A: Found. Crystallogr*. **2008**, *64*, 112.
